# Efficient synthesis of antiviral agent uprifosbuvir enabled by new synthetic methods†

**DOI:** 10.1039/d1sc01978c

**Published:** 2021-05-19

**Authors:** Artis Klapars, John Y. L. Chung, John Limanto, Ralph Calabria, Louis-Charles Campeau, Kevin R. Campos, Wenyong Chen, Stephen M. Dalby, Tyler A. Davis, Daniel A. DiRocco, Alan M. Hyde, Amude M. Kassim, Mona Utne Larsen, Guiquan Liu, Peter E. Maligres, Aaron Moment, Feng Peng, Rebecca T. Ruck, Michael Shevlin, Bryon L. Simmons, Zhiguo Jake Song, Lushi Tan, Timothy J. Wright, Susan L. Zultanski

**Affiliations:** Department of Process Research and Development, Merck & Co., Inc. Rahway New Jersey 07065 USA artis_klapars@merck.com; WuXi STA 90 Delin Road, Waigaoqiao Free Trade Zone Shanghai 200131 China

## Abstract

An efficient route to the HCV antiviral agent uprifosbuvir was developed in 5 steps from readily available uridine in 50% overall yield. This concise synthesis was achieved by development of several synthetic methods: (1) complexation-driven selective acyl migration/oxidation; (2) BSA-mediated cyclization to anhydrouridine; (3) hydrochlorination using FeCl_3_/TMDSO; (4) dynamic stereoselective phosphoramidation using a chiral nucleophilic catalyst. The new route improves the yield of uprifosbuvir 50-fold over the previous manufacturing process and expands the tool set available for synthesis of antiviral nucleotides.

## Abstract 2

An efficient route to the HCV antiviral agent uprifosbuvir was developed in 5 steps from readily available uridine in 50% overall yield.

## Graphical abstract

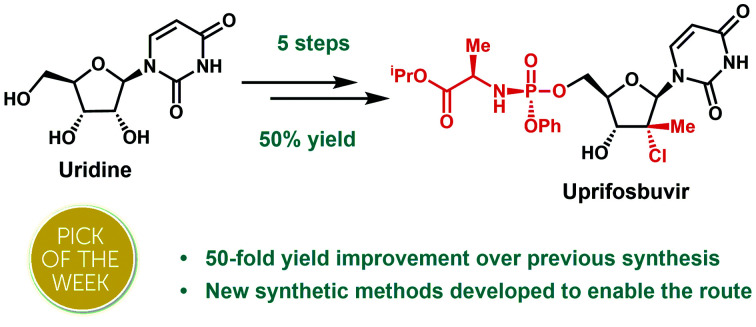

## Introduction

As highlighted by the COVID-19 pandemic, availability of efficient antiviral treatments remains a starkly unmet medical need for most viral infections. Among existing antiviral drugs, several have been approved for the treatment of more than one viral disease^1^ and already approved antiviral drugs can sometimes be repurposed as treatments for emerging infectious diseases.^2^ This potential for emergency use underscores the need for a large and diverse stockpile of antiviral agents and the importance of efficient manufacturing processes to enable a rapid response to a potentially massive increase in demand.

Chronic hepatitis C virus (HCV) infection affects an estimated 71 million people globally, and the WHO has estimated that in 2016 approximately 400 000 people died from HCV-related complications.^3^ Recently, encouraging progress has been achieved in the treatment of HCV with cure rates now exceeding 95%.^4^ Uprifosbuvir **1** is an NS5b inhibitor developed for the treatment of HCV,^5^ representing a class of 2′-branched nucleosides modified with a ProTide sidechain.^6^ Many other nucleoside antivirals also contain 2′, 3′, or 4′-modifications of the ribose core, presenting formidable synthetic challenges that have been approached through *de novo* synthesis.^5,7^ The alternative and more direct strategy to functionalize preexisting nucleosides is less developed.^8^ Here, we report a highly efficient synthesis of uprifosbuvir **1***via* direct functionalization of uridine enabled by development of new synthetic methods to overcome limitations of the existing tool set available for the synthesis of nucleotides.

## Results and discussion

**Scheme 1:**
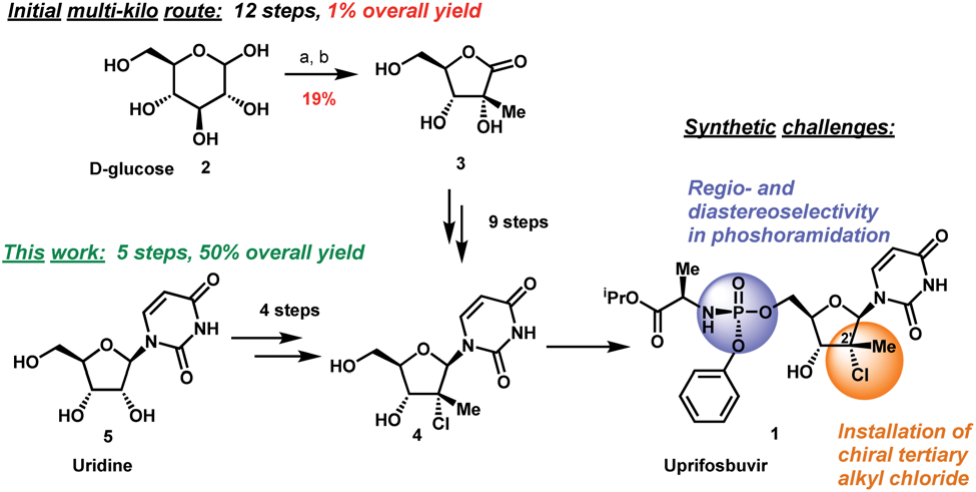
Synthetic approaches to uprifosbuvir **1** with the two main challenges highlighted. (a) Me_2_NH, AcOH, EtOH/MeOH, 80 °C, 1.5 h; (b) Ca(OH)_2_, water, 70 °C, 24 h, 19% over 2 steps.^9^

The original multi-kilogram route to uprifosbuvir proceeded in 12 linear steps and only 1% overall yield (Scheme 1). Commencing with d-glucose **2**, the route utilized an ingenious albeit low-yielding (19%) rearrangement cascade to reach lactone **3** containing the tertiary alkyl group at the 2′-position.^9^ Further elaboration of lactone **3** over 10 steps provided uprifosbuvir **1**. In addition to the challenges in stereoselective installation of the chiral tertiary alkyl chloride, the route suffered from low yields and poor regio- and diastereoselectivity in phosphoramidation of intermediate **4** to install the ProTide sidechain (Scheme 1). A more efficient synthesis was urgently required to address these two key synthetic challenges and to meet the antiviral therapy demand.

**Scheme 2:**
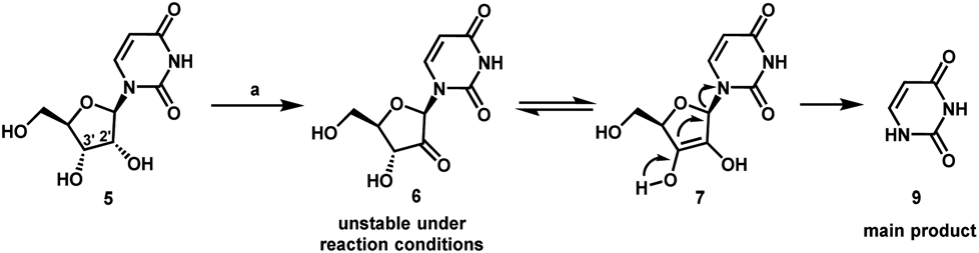
Decomposition during attempted direct biocatalytic oxidation of uridine **5**. (a) General screening conditions: KRED enzyme variants, NAD or NADP cofactor, acetone-water, 30 °C. The reactions were screened using a set of buffers with pH of 6.5–7 (potassium phosphate) and pH of 5–6 (sodium borate).

We envisioned a shorter and more efficient route to uprifosbuvir starting from the inexpensive and readily available uridine **5**,^10^ which already contains all of the atoms in the nucleoside portion of the molecule, with the glaring – and challenging – exception of the tertiary alkyl chloride in the 2′-position. We started out by exploring selective functionalization in the 2′-position in uridine. All chemical^11^ or biocatalytic efforts to directly oxidize uridine failed due to poor selectivity or instability of 2′-ketouridine **6**. Uracil **9** was the main product observable by HPLC in the biocatalytic oxidation using ketoreductase (KRED) enzymes in the reverse direction, presumably formed *via* fast enolization of 2′-ketouridine **6** (or the isomeric 3′-ketouridine) to **7** followed by elimination of uracil (Scheme 2).^12^

It became clear that a minimal protecting group strategy for uridine would be required to address the aforementioned instability, as well as solubility, issues. Protection of the 3′,5′-diol moiety in riboses is traditionally achieved with 1,3-dichloro-1,1,3,3-tetraisopropyl-disiloxane (TIPDSCl_2_)^8,13^ or di-*tert*-butylsilyl ditriflate (DTBS(OTf)_2_).^14^ Although these protecting reagents are selective for 3′,5′-diprotection, they are expensive and have high molecular weights, rendering them impractical for commercial manufacturing. In order to better accomplish our goals for the synthesis of uprifosbuvir, we decided to evaluate simple acyl chlorides as far more practical protecting groups.

**Scheme 3:**
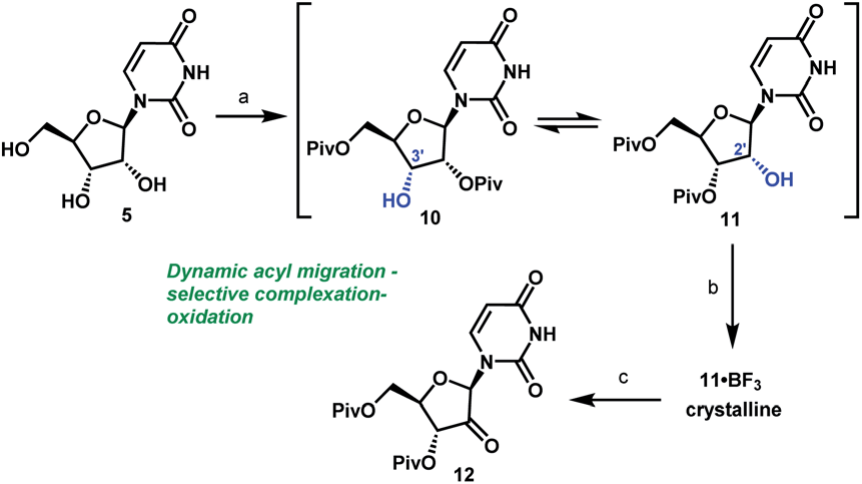
Complexation-driven selective acyl migration/oxidation to access **12**. (a) PivCl, pyridine, 0 °C, 16 h; (b) BF_3_·OEt_2_, PhMe, 40 °C, 10 h; (c) TEMPO, Bu_4_NBr, AcOOH, dioctyl sulphide, PhMe, −10 °C to 20 °C, 24 h, 83% from **5**.

Unsurprisingly, acylation of uridine provided mixtures of products, including a series of mono-, bis-, and tri-acylated products. Of several simple acyl chlorides that were evaluated,^15^ pivaloyl chloride (PivCl) delivered the highest selectivity for diacylation (>90%) *vs.* mono- and tri-acylation; therefore, PivCl was selected as the acylating agent for further optimization. Although the kinetic ratio favoured the undesired 2′,5′-isomer **10** (87 : 13 of **10**/**11** at 0 °C), the two isomers could be equilibrated to a 1 : 3.3 thermodynamic ratio of **10**/**11** (Scheme 3). We then investigated the strategy of influencing this equilibrium through selective complexation of **11** with a Lewis acid. This could be followed by oxidation to the desired ketone **12** in a complexation-driven selective acyl migration/oxidation process. Carrying out a high throughput screen of 96 Lewis acidic additives led to rapid identification of BF_3_·OEt_2_ as a unique additive that afforded >99 : 1 selectivity of **11**/**10** in toluene solvent. The likely driving force for this isomerization is formation of the crystalline **11·BF3** complex.^16^ This hypothesis is supported by our observations that selective isomerization was not observed in alternative solvents, such as THF, MeCN, EtOAc that are more polar and Lewis basic towards BF_3_ than toluene and could potentially compete with **11** or dissolve the **11·BF3** complex. The slurry of crystalline **11·BF3** complex (>50 : 1) in toluene could be treated with water to dissolve the crystals and provide a slightly acidic organic layer (pH = 2) that maintained its enrichment in **11** (>50 : 1) due to very slow rate of equilibration of **10** and **11** under mildly acidic conditions. For the subsequent oxidation step, the improved stability of both alcohol **11** and ketone **12** at low pH led us to select the mildly acidic TEMPO/Bu_4_NBr/AcOOH conditions. Direct isolation of crystalline ketone **12** from the reaction mixture provided 83% yield from uridine **5** with >50 : 1 selectivity (Scheme 3). In this way, the unfavorable kinetic selectivity of 13 : 87 (**11**/**10**) in the pivaloylation of **5** could be inverted to >50 : 1 selectivity in the formation of ketone **12** using a novel complexation-driven selective acyl migration/oxidation.

**Scheme 4:**
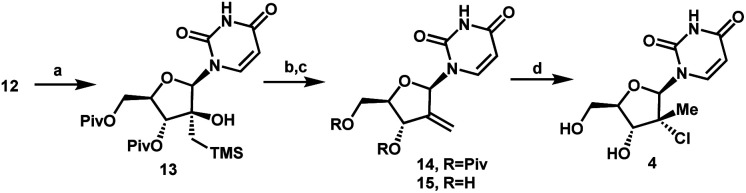
Olefination/hydrochlorination of the ketone **12**. (a) TMSCH_2_MgCl, CPME, 0 °C to rt, 24 h, 91%; (b) (CF_3_CO)_2_O, pyridine, DMAP, MeCN, rt, 15 h, then KF, 70 °C, 24 h, 88%; (c) K_2_CO_3_, MeOH/THF, 40 °C, 15 h, 94%; (d) FeCl_3_, PhSiH_3_, rt, 48 h, 82%.

With a practical process for the preparation of 2′-ketone **12** achieved, we explored means to introduce the methyl and chloro substituents in the 2′-position. First, we investigated a sequence of olefination/hydrochlorination. Although Wittig olefination had been reported for a related ketone where TIPDS protecting group was used instead of Piv protecting groups,^17^ only low yields of olefin **14** were obtained from ketone **12** under a variety of conditions. We consequently moved on to explore Peterson olefination (Scheme 4). The β-hydroxysilane **13** could be formed in high yield upon treatment of ketone **12** with Me_3_SiCH_2_MgCl. Elimination to olefin **14** was challenging under either basic or acidic conditions; nevertheless, we were able to achieve it through activation of the tertiary alcohol as a trifluoroacetate ester followed by fluoride-mediated desilylation/elimination to provide the desired olefin **15**. Unfortunately, direct hydrochlorination of olefin **15** with various HCl sources failed to afford the desired tertiary alkyl chloride **4**. Instead, decomposition of **15** was observed, as evidenced by the formation of uracil **9**. In our search for milder hydrochlorination conditions, we were intrigued by a single example in a publication by Boger and co-workers where Fe(iii) oxalate, 4-AcNHC_6_H_4_SO_2_Cl, and NaBH_4_ were used to effect the hydrochlorination of a simple alkene.^18^ Applying these conditions to **15** led to formation of the desired alkyl chloride **4** in 6% yield. Further development revealed FeCl_3_ and PhSiH_3_ as much more efficient reagents; moreover, a sulfonyl chloride was not required. Under these improved and streamlined conditions, olefin **15** could be converted into chloride **4** at rt with high diastereoselectivity (>50 : 1), 95% assay and 82% isolated yield. This novel olefination/hydrochlorination sequence allowed us to install the two critical methyl and chloro functionalities in 3 steps starting from ketone **12**.

Although the olefination/hydrochlorination route was a major improvement over the previous synthesis, we were not satisfied with the relatively high cost and poor availability of the Me_3_SiCH_2_MgCl reagent and unfavorable process mass intensity inherent to most olefination reactions. Therefore, we desired to define an alternative route with comparable step count. For the introduction of the methyl group, leveraging methylmagnesium reagents was particularly attractive due to their low cost and excellent availability. Under all conditions evaluated, methyl addition to ketone **12** favoured the α-facial attack, although the selectivity was modest (Table 1, entries 1 and 2). With three equivalents of the Grignard reagent, >94% conversion was achieved, but the reactions suffered from poor yields of the desired tertiary alcohol **16** due to competitive cleavage of the pivaloyl ester. In order to suppress these side reactions, we evaluated milder methyl nucleophiles. Using 3.3 equiv. of Me_2_AlCl, the reaction proceeded to 98% conversion with 49 : 1 dr and 86% yield and completely avoided the de-Piv side reaction (Table 1, entry 3). However, due to the highly hazardous nature of alkylaluminums, we continued to look for a more practical reagent for this transformation. Given the attractiveness of utilizing a Grignard reagent from a cost, safety and availability perspective, we hypothesized that the presence of Lewis acid additives could attenuate the reactivity of MeMgBr and offset the competitive undesired reactions. Favourable results were obtained in the presence of ZnCl_2_, which afforded 91% yield with 21 : 1 dr; however, 4 equiv. of MeMgBr and 2 equiv. of ZnCl_2_ were required to drive the reaction to 98% conversion, leading to cost and environmental concerns (entry 4). We ultimately identified that using the slightly more reactive organomanganese reagent,^19^ prepared from 2.5 equivalents each of MeMgBr and MnCl_2_, afforded high yield and high dr of the desired methylated product. Changing the solvent to anisole/toluene led to further improvement in the dr (entries 5 and 6). These conditions provide the ideal solution for installation of the methyl moiety of uprifosbuvir.

**Table 1:** Selective methyl addition to ketone **12**

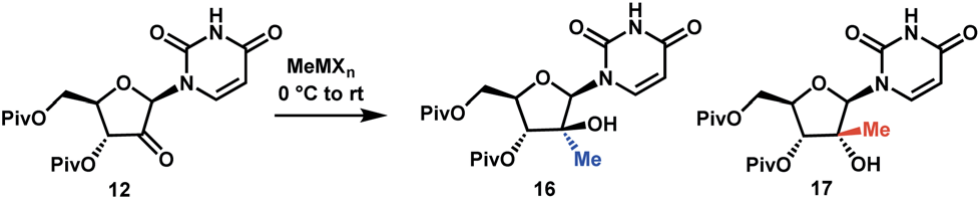						
Entry	Methyl nucleophile	Equiv.	Solvent	d.r. (**16** : **17**)	De-Piv^a^ (%)	AY (%) of **16**^b^
1	MeMgBr	3.0	CH_2_Cl_2_	10 : 1	14	62
2	MeMgCl	3.0	CH_2_Cl_2_	5 : 1	40	—
3	Me_2_AlCl	3.3	CH_2_Cl_2_	49 : 1	—	86
4	MeMgBr/ZnCl_2_	4/2	PhMe/2-MeTHF	21 : 1	—	91
5	MeMgBr/MnCl_2_	2.5/2.5	PhMe/2-MeTHF	12 : 1	—	92
6	MeMgBr/MnCl_2_	2.5/2.5	PhMe/anisole	32 : 1	—	93

a Deprotection of Piv group, as indicated by the sum of mono-Piv derivatives of **16** and **17**.

b Assay yield after work-up.

**Scheme 5:**
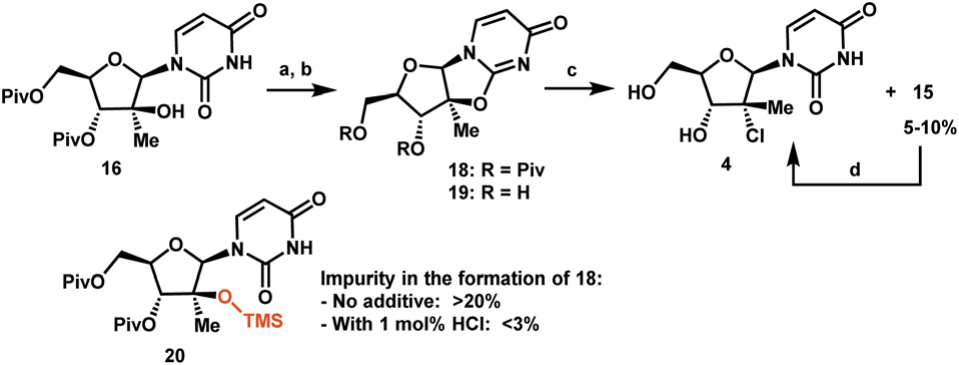
Formation of tertiary chloride **4**. (a) BSA, 1 mol% of 37% aq HCl, anisole, 75 °C, 8 h; (b) DBU, MeOH, 60 °C, 10 h, 87% yield from **16**; (c) Me_2_SiCl_2_, DMF, 1,2-dimethoxyethane (DME), 70 °C, 10 h; (d) FeCl_3_–6H_2_O, (Me_2_SiH)_2_O, 25 °C, 12 h, 85% from **19**.

Having introduced the methyl group in the 2′-position, it was necessary to convert the tertiary alcohol **16** into the corresponding tertiary chloride **4** with concomitant inversion of configuration. Carrying out this transformation on such densely functionalized molecule as **16** presents a series of challenges: (1) selectivity among the OH and ester groups, (2) risk of elimination from the tertiary alcohol, (3) diastereoselectivity of displacement at the 2′-position, and (4) intramolecular reactivity of neighbouring groups. Attempts at direct conversion of alcohol **16** into chlorouridine **4** resulted in complex mixtures with low yields of the desired product **4**. Therefore, we decided to take advantage of the neighbouring uracil ring to form the desired chloride **4***via* anhydrouridine **19**^20,21^ with overall inversion of configuration at the 2′-position (Scheme 5). First, we needed to form anhydrouridine **18** with retention of configuration at 2′-position of alcohol **16**, which was unprecedented and required selective activation of the uracil ring in the presence of the 2′-alcohol.^22^ We screened various activating agents and found that bis-trimethylsilylacetamide (BSA) promoted cyclodehydration of **16** to provide **18**. Unfortunately, a significant amount of *O*-TMS derivative **20** was also formed as an impurity (>20% assay yield) that failed to convert to the desired **18** under the reaction conditions. We speculated that the formation of TMS ether impurity **20** was likely base-catalyzed and were delighted to find that addition of 1 mol% HCl suppressed the TMS ether impurity **20** to <3%. Following cyclodehydration, the pivalate protecting groups were conveniently removed in the same reaction vessel by addition of DBU and MeOH, and the crystalline deprotected anhydrouridine **19** was directly isolated from the reaction mixture in 87% isolated yield, thereby positioning us for installation of the key chloro functionality.

Formation of the tertiary chloride **4** from **19** is challenging due to the presence of considerable steric hindrance and vicinal electron-withdrawing substituents. The initial conditions developed were highly hazardous (HCl gas, AcOH, 1,4-dioxane, under pressure at 100 °C) and provided only 56% yield of **4** from **19**. Milder and more convenient conditions were established by using dichlorodimethylsilane to form a more soluble silyl ether intermediate and generate HCl *in situ*.^23^ However, under these and many other conditions explored, 5–10% of olefin **15** was consistently formed as an impurity. This outcome not only served to reduce the yield of the desired chloride **4** but also complicated the isolation and purification of **4** by crystallization due to low solubility of the olefin impurity **15**. Having previously developed a highly efficient Fe-mediated hydrochlorination of olefin **15** (Scheme 4), we employed this innovation and were delighted to find that, simply adding the inexpensive reagents FeCl_3_·6H_2_O and tetramethyldisiloxane (TMDSO) at the end of the dichlorodimethylsilane-mediated hydrochlorination of **19** cleanly funnelled the olefin impurity **15** into tertiary alkyl chloride **4** with high diastereoselectivity (>100 : 1 overall dr of **4**).^24^ In so doing, we carried out a two-step sequence to successfully convert a sterically encumbered tertiary alcohol into the corresponding inverted tertiary chloride and reveal the full nucleoside architecture of uprifosbuvir.

**Scheme 6:**
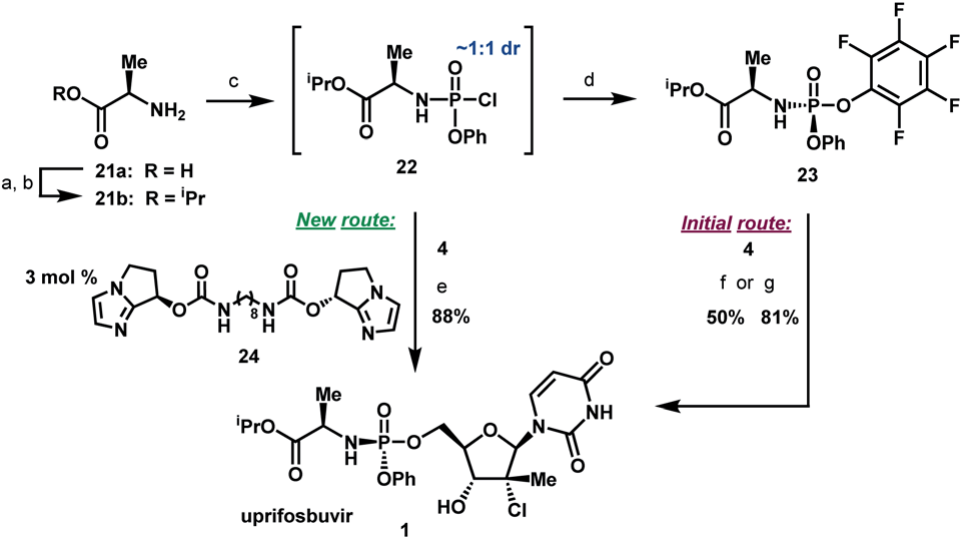
Completion of uprifosbuvir synthesis. (a) TMS-Cl, iPrOH, 70 °C, 12 h; (b) NEt_3_, iPrOAc, wiped film evaporation, 80%; (c) PhOP(O)Cl_2_, NEt_3_, iPrOAc, −20 °C, 2 h, 90%; (d) C_6_F_5_OH, NEt_3_, iPrOAc, −5 °C to 10 °C, 18 h, 76%;^26^ (e) **4**, 3 mol% **24**, 2,6-lutidine, 1,3-dioxolane, −10 °C, 24 h, 88%; (f) **4**, tBuMgCl, THF, −5 °C to 5 °C, 15 h, 50%;^27^ (g) **4**, Me_2_AlCl, 2,6-lutidine, THF, 35 °C, 16 h, 81%.^27^

To complete the synthesis of uprifosbuvir, it remained to install the phosphoramidate side chain, presenting a significant synthetic challenge. The initial kilo-scale synthesis proceeded through the diastereopure pentafluorophenyl ester **23**^25,26^ and utilized *t*BuMgCl as base to provide uprifosbuvir **1** with poor 3′/5′ regioselectivity and only 50% isolated yield. We found that introducing Me_2_AlCl as an additive led to a significant improvement in the phosphoramidation regioselectivity, resulting in an 81% isolated yield of uprifosbuvir.^27^ Nevertheless, this approach still required preparation of the enantio and diastereopure phosphorylating agent **23**. An even more efficient route would bypass **23** altogether and would directly introduce the phosphoramidate sidechain in **4** using chlorophosphoramidate **22**, which can be readily prepared from alanine ester **21b** and conveniently used as a solution in iPrOAc.^28^ However, chlorophosphoramidate **22** exists as a ∼1 : 1 mixture of diastereomers and uncatalyzed phosphoramidation of **4** with **22** was shown to proceed to uprifosbuvir in poor diastereoselectivity (55 : 45 dr at phosphorus). In an earlier communication,^29,30^ we disclosed a class of dimeric chiral imidazole carbamate catalysts that was highly effective in overcoming the unfavourable inherent diastereoselectivity in dynamic stereoselective phosphoramidation of nucleosides. Upon further development, we identified **24** as the optimal catalyst for **1** and found that the crude chlorophosphoramidate mixture **22** (1 : 1 dr) could be coupled with **4** in 97 : 3 dr and 94% assay yield using only 3 mol% of catalyst **24**, providing 88% isolated yield of uprifosbuvir **1** after crystallization. This highly efficient dynamic stereoselective phosphoramidation step concluded the 5-step synthesis of uprifosbuvir in 50% overall yield from uridine (Scheme 6).

## Conclusions

**Scheme 7:**
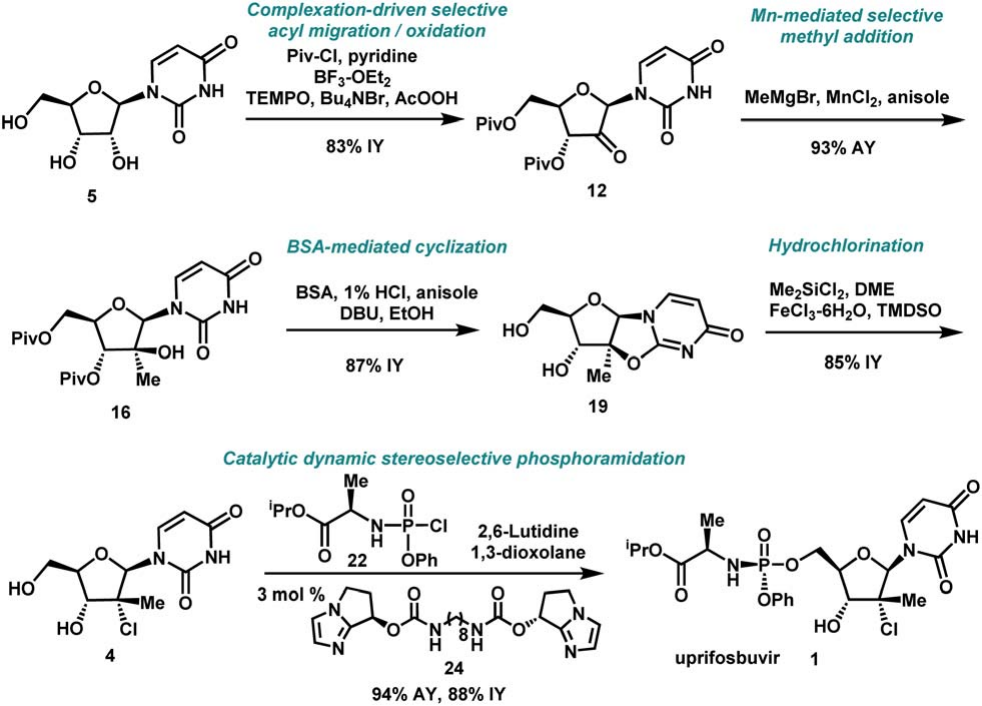
Summary of uprifosbuvir synthesis. AY = assay yield; IY = isolated yield.

In summary, we have developed a highly efficient route to HCV antiviral uprifosbuvir **1** in five easily scalable steps from the readily available raw material uridine **5** in 50% overall yield, which represents a 50-fold yield improvement over the initial multi-kilo route. This achievement was made possible by the development of several synthetic methods: (1) complexation-driven selective acyl migration/oxidation; (2) BSA-mediated cyclization to anhydrouridine; (3) hydrochlorination using Me_2_SiCl_2_ and FeCl_3_/TMDSO; (4) dynamic stereoselective phosphoramidation using a chiral nucleophilic imidazole carbamate catalyst (Scheme 7).

Nucleoside antiviral agents for various indications often share common structural features, such as ribose modifications and the ProTide moiety. We believe that the methods disclosed here for the synthesis of uprifosbuvir should facilitate more efficient preparation of other nucleoside antivirals to enable access to these life-saving medicines.^31^

## Author contributions

A. K., J. Y. L. C., J. L., R. C., W. C., S. M. D., T. A. D., D. A. D., A. M. H., A. M. K., M. U. L., P. E. M., A. M., F. P., M. S., B. L. S., T. J. W. and S. L. Z. conceived and performed the experiments; A. K., J. L., L.-C. C., K. R. C., M. U. L., G. L., A. M., R. T. R. and L. T. led the project and conceived ideas; A.K. and J. Y. L. C. wrote the original draft; L.-C. C., A. M. H. and R. T. R. reviewed and edited the manuscript; all authors contributed to the discussion of the results and the final manuscript.

## Conflicts of interest

There are no conflicts to declare.

## Supplementary Material

SC-012-D1SC01978C-s001
