# Supplementary material for: Efficient synthesis of antiviral agent uprifosbuvir enabled by new synthetic methods
Source: Chem Sci. 2021 May 19;12(26):9031–6. doi: 10.1039/d1sc01978c (PMC8261776; doi:10.1039/d1sc01978c)
Supplement: SC-012-D1SC01978C-s001 [file SC-012-D1SC01978C-s001.pdf]

Electronic Supplementary Material (ESI) for Chemical Science.
This journal is © The Royal Society of Chemistry 2021

Efficient Synthesis of Antiviral Agent Uprifosbuvir Enabled by New Synthetic Methods

Artis Klapars,* John Y. L. Chung,* John Limanto, Ralph Calabria, Louis-Charles Campeau, Kevin R. Campos, Wenyong Chen, Stephen M. Dalby, Tyler A. Davis, Daniel A. DiRocco, Alan M. Hyde, Amude M. Kassim, Mona Utne Larsen, Guiquan Liu, Peter E. Maligres, Aaron Moment, Feng Peng, Rebecca T. Ruck, Michael Shevlin, Bryon L. Simmons, Zhiguo Jake Song, Lushi Tan, Timothy J. Wright and Susan L. Zultanski

Supporting Information

Experimental Procedures

General Techniques. All reagents and solvents were obtained from commercial sources and used as received. All reactions were performed under an atmosphere of nitrogen with unpurified reagents and dry solvents. Analytical thin-layer chromatography (TLC) was performed using 0.25 mm silica gel coated plates. Flash chromatography was performed using the indicated solvent and silica gel 60 (230-400 mesh). ^{1}H NMR spectra (400 and 500 MHz) and ^{13}C NMR spectra (100 and 125 MHz) were recorded on Bruker spectrometers in the indicated solvent. Chemical shifts (δ) are reported in units of parts per million (ppm) relative to the signal for internal standards. High resolution mass spectra were recorded in ESI mode using magnetic sector mass analyzer and TOF mass spectrometer.

Preparation of ketone 12 from uridine 5. To a dry vessel equipped with overhead stirrer under N_2 atmosphere was charged anhydrous pyridine (305 kg, < 0.05 wt% H_2O) and uridine (100.8 kg, 412.7 mol). After agitating at 20-25 °C for at least 1 h to dissolve solids, the temperature was adjusted to 0-5 °C, then 2.2 equiv pivaloyl chloride (109 kg, 904 mol) was slowly added while maintaining the temperature at 0-10 °C. After finishing the addition, the temperature was adjusted to 20-25 °C and the reaction was stirred for 14-24 h at 20-25 °C. The reaction was monitored by HPLC: if (uridine + mono-Piv uridine)/(**10**+**11**) ≤ 4%, proceeded to aqueous work up, if ≥ 4%, added 0.01-0.2 equiv of pivaloyl chloride at 15-20 °C over 30-60 min, then stirred for 5-8 h at 20-25 °C. H_2O (400 kg) was added while keeping temperature between 10-30 °C. The mixture was stirred at 40 °C for 16-20 h. The progress was checked by HPLC, targeting Tris-Piv species to < 0.2 A% each, and **11/10** ~ 2:1. The batch was cooled to 20-25 °C, then toluene (437 kg) was added. The layers were separated and the aqueous layer discarded. The organic layer was washed with 2.5 N HCl (866 kg). To the organic layer was charged 2-methyl-THF (214 kg) and the resulting batch was adjusted to 25-30 °C, then washed with 5% $NaHCO_3$ (517 kg). After discarding the aqueous layer, the organic phase was washed with H_2O (510 kg), while keeping batch temperature between 25-30 °C. The batch was concentrated by distillation under reduced pressure until vessel achieved a 5X relative to uridine by volume toluene solution. The distillation was continued with slowly feeding ~7.5X by volume toluene (~ 765 L) and distilling at a constant volume of 5X by volume until met targets of < 300 ppm pyridine, < 300 ppm water and ≤ 0.25 wt% 2-methyl-THF. Typical **10/11** concentration was 300-330 mg/g with a assay yield of 90-95% as a ~1:2 mixture. The impurities were typically

0-2% LCAP of mono-piv uridine and 3-5% LCAP tris-piv uridine. Although chemically stable, the toluene solutions may begin to show signs of solid precipitation if held over several days. The precipiated solid was pure **10**. Analytical samples of **10/11** were obtained via flash chromatography purification (EtOAc/hexanes) and characterized as a mixture. Compound **10**: ^{1}H NMR ($CDCl_3$, 500 MHz): δ 9.99 (s, NH), 7.42 (d, *J* = 8.1 Hz, 1H), 5.9 (d, *J* = 3.7 Hz, 1H), 5.70 (d, *J* = 8.2 Hz, 1H), 5.18 (dd, *J* = 5.6, 3.8 Hz, 1H), 4.24-4.40 (m, 3H), 4.16 (dd, *J* = 6.8, 4.2, 2.8 Hz, 1H), 3.71 (d, *J* = 4.5 Hz, OH), 1.19 (s, 18H); ^{13}C NMR ($CDCl_3$, 125 MHz,): δ 178.25, 177.97, 163.72, 150.21, 140.12, 102.74, 88.53, 81.60, 75.33, 69.09, 63.38, 38.92, 38.81, 27.17, 27.04; HRMS $[M+H]^+$ for $C_{19}H_{29}N_2O_8$ calc'd 413.1924; found 413.1928. Compound **11**: ^{1}H NMR ($CDCl_3$, 500 MHz): δ 10.25 (s, NH), 7.49 (d, *J* =8.2 Hz, 1H), 5.87 (d, *J* = 4.8 Hz, 1H), 5.70 (d, *J* = 8.2 Hz, 1H), 4.93 (t, *J* = 5.2 Hz, 1H), 4.54 (d, *J* = 4.8 Hz, OH), 4.24-4.40 (m, 4H), 1.17 (s, 18H) . ^{13}C NMR ($CDCl_3$, 125 MHz,): δ 177.94, 177.92, 163.62, 150.93, 139.29, 102.81, 89.83, 79.97, 73.51, 71.09, 63.07, 38.86, 38.81, 27.15, 27.00; HRMS $[M+H]^+$ for $C_{19}H_{29}N_2O_8$ calc'd 413.1924; found 413.1917. HPLC method: Ascentis Express C18, 4.6×150 mm, 2.7 μm; B = CH_3CN, A = 0.1% H_3PO_4 in H_2O; 10%B to 90%B over 10 min, hold at 90%B for 2 min, posttime = 3 min; flow rate = 1.0 mL/min; injection volume = 5 μL; detection = 261 nm; retention times: 4.46 min & 4.90 min = mono-pivalolylated uridines, 7.46 min = 3',5'-O-dipivaloyluridine, 7.95 min = 2',5'-O-dipivaloyluridine, 10.56 min & 10.62 min = tris-pivaloylated uridines). **Part II - Isomerization of 10/11 mixture *via* BF_3 complex of 11.** To a vessel under N_2 was charged **10/11** toluene solution from above (275 L×305 mg/mL = 83.8 kg, 203.3 mol), followed by 6X by volume toluene (364 L) to adjust to a 12X by volume toluene solution relative to uridine (or 7X by volume relative to bis-Piv uridines at 130-150 mg/g). The batch temperature was adjusted to 20-25 °C, then 1.22 equiv $BF_3·OEt_2$ (35.2 kg, 248 mol) was charged over > 30 min. The resultant slurry was aged at 40 °C for 10 h, then cooled to 20-25 °C (**11** selectively formed a complex with BF_3 and precipitated from the reaction mixture. The formation of this complex was inhibited by residual water and pyridine. Both must be individually controlled below 300 ppm; Target ratio of **11/10** > 98 : 2). After adjusting the batch temperature to 0-10 °C, H_2O (385 kg) was charged, then stirred at 0-10 °C for 1 h. The mixture was allowed to settle, then separated and the aqueous bottom layer discarded. Again, to the organic layer was charged H_2O (385 kg), stirred at 0-10 °C for 1 h, then settled and cut and discarded the aqueous bottom layer. Typical yield of **11** in the organic phase was >99% for the step or >90% from uridine. The bis-Piv-uridine **11/10** solution will maintain > 98 : 2 ratio upon storage for at least 4 weeks when held at 0-5 °C. Typical water content of this solution is ~0.4 wt%. Typical concentration of **11** solution at this point is 130-145 mg/g, which would serve as the basis for all charges in the oxidation. **Part III - TEMPO/AcOOH Oxidation of 11 to ketone 12**. To a vessel was charged bis-Piv-uridine **11** solution from above (assayed 38.4 kg, 93.1 mol in a 13.5 wt% solution), then adjusted the temperature of the content to 0-5 °C. To a second vessel was charged 3.3X by volume toluene (127 L), adjusted temperature to 20-25 °C and initated a sufficient N_2 cross-sweep so as to keep O_2 composition within a safe operation level. To the second vessel was charged 0.166 equiv tetrabutylammonium bromide (TBAB) (4.98 kg, 15.45 mol), followed by 0.25 equiv TEMPO (3.6 kg, 23.3 mol), then aged for 15-30 min to make sure that TEMPO dissolved, then cooled the mixture to -10 °C. TBAB is only partially soluble in the reaction initially but completely dissolved as the reaction progressed. 1.5 equiv 40 wt% peracetic acid (27.2 kg, 143 mol) at 5 °C was added to the second vessel over 12-16 h at -10 °C. After 3.6% of the total AcOOH was charged (over 30 min), stopped the addition and aged the contents for 1 h. The remainder AcOOH

and the **11** solution from the first vessel was charged simultaneously over 13.5 h at -10 °C, then rinsed with 12 L toluene. The batch was aged at -10 °C for 3 h, then sampled for reaction completion. If the reaction conversion is < 97%, aged the batch for additional 5 h. If conversion is > 97% proceeded to charge 0.332 equiv di-*n*-octylsulfide (8 kg, 30.9 mol) at -10 °C to quench any remaining peracetic acid. The reaction slurry was stirred for 0.5 h, warmed the batch to 0 °C and aged for 2-3 h at 0 °C until hydrogen peroxide < 100 ppm. The batch temperature was adjusted to 20 °C, and the reaction slurry was stirred for 2-3 h until ≤ 8 mg/g ketone **12** in filtered supernatant. The slurry mixture was filtered, and the cake was displacement washed with toluene (77 L) and water (77 L). The cake was slurry washed with a combined mixture of toluene (58 L) and water (58 L) for 1 h with slow agitation (4-8 rpm) before filtering. The cake was displacement washed with water (77 L), and toluene (77 L), then the wet cake was dried under vacuum at 30-35 °C for 12-48 h. Isolated yield of **12** was 34.2 kg (90% yield from **11**, 83% yield from **5**) with > 98% LCAP purity, containing < 0.2 wt% water, < 890 ppm toluene and < 5000 ppm acetic acid. Both the keto and keto-hydrate forms were observed by reversed phase LC/MS, whereas only the keto form was observed in $CDCl_3$ by NMR. Compound **12**: ^{1}H NMR ($CDCl_3$, 500 MHz): δ 11.71 (s, NH), 7.85 (d, *J* = 8.0 Hz, 1H), 5.76 (s, 1H), 5.72 (d, *J* = 7.9 Hz, 1H), 5.31 (d, *J* = 7.3 Hz, 1H), 4.37 (dt, *J* = 7.3, 5.2 Hz, 1H), 4.31 (dd, *J* = 11.8, 4.6 Hz, 1H), 4.23 (dd, *J* = 11.8, 5.4 Hz, 1H), 1.19 (s, 9H), 1.15 (s, 9H); ^{13}C NMR ($CDCl_3$, 125 MHz,): δ 201.96, 177.13, 177.04, 163.14, 150.22, 144.69, 102.39, 84.40, 75.93, 70.68, 62.97, 38.31, 38.06, 26.78, 26.54; HRMS $[M+H]^+$ for $C_{19}H_{27}N_2O_8$ calc'd 411.1767; found 411.1765. Hydrated **12** (observed only in LC): HRMS $[M+H]^+$ for $C_{19}H_{29}N_2O_9$ calc'd 429.1873; found 429.1884.

Preparation of β-hydroxysilane 13 from ketone 12. A solution of ketone **12** (6.25 g, 1 equiv) in cyclopentyl methyl ether (CPME) (30 mL) was cooled to 0 °C, and to the cooled solution was added a 1 M solution of $TMSCH_2MgCl$ (42.6 mL, 2.5 equiv) in diethyl ether over a 15-30 min period. The resulting reaction was allowed to stir overnight at room temperature, then 2N HCl was then added. The resulting reaction was allowed to stir for 10 min at room temperature, the organic layer was separated and washed with water, brine, dried over $MgSO_4$, filtered, concentrated, and purified by silica gel chromatography (hexane-ethyl acetate) to provide 6.90 g (91% yield) of alcohol **13** as foamy solid. ^{1}H NMR ($CDCl_3$, 500 MHz): δ 9.07 (s, 1H), 7.66 (d, *J* = 8.2 Hz, 1H), 6.07 (s, 1H), 5.66 (dd, *J* = 8.2, 2.0 Hz, 1H), 4.76 (s, 1H), 4.70 (dd, *J* = 11.8, 7.1 Hz, 1H), 4.31 (dd, *J* = 11.8, 3.9 Hz, 1H), 4.02-4.07 (m, 1H), 3.40 (s, 1H), 1.21-1.31 (m, 19H), 1.15 (d, *J* = 15.3 Hz, 1H), 0.04 (s, 9H). ^{13}C NMR ($CDCl_3$, 125 MHz,): δ 178.16, 177.27, 163.53, 150.96, 142.63, 101.37, 90.13, 82.45, 81.41, 78.74, 63.86, 38.87, 38.75, 27.13, 27.08, 19.59, 0.13. HRMS $[M+H]^+$ for $C_{23}H_{38}N_2O_8Si$ calc'd 499.2476; found 499.2474.

Preparation of olefin 15 from β-hydroxysilane 13. To a solution of alcohol **13** (1 g, 1 equiv) in dry acetonitrile was added trifluoroacetic anhydride (0.75 mL, 3 equiv), pyridine (0.48 mL, 3 equiv) and DMAP (49 mg, 0.2 equiv) and the resulting reaction was allowed to stir at room temperature for 15 h. Potassium fluoride (0.45 g, 3.5 equiv) was then added and the resulting reaction was heated to 70 °C and allowed to stir at this temperature for 24 h. MTBE was then added, followed by H_2O. The organic layer was separated and washed with another portion of water, followed by an aqueous solution containing 2 equiv of K_2CO_3 and brine. The final organic layer was concentrated in vacuo to provide crude olefin **14** (720 mg, 88% yield) as a solid. Compound **14** (0.30 g, 1 equiv) was dissolved in a mixture of MeOH (1 mL) and THF (1 mL), treated with K_2CO_3 (0.305 g, 3 equiv) and allowed to age for 20 h at 40 °C. The reaction was

then concentrated *in vacuo* and solvent switched to 2-MeTHF (6 mL) and treated with 4M HCl (2 equiv) in dioxane. The resulting slurry was concentrated *in vacuo* to half of its volume, filtered, and the filtrate was concentrated to provide 165 mg (94% yield) of diol **15** as a white solid. The NMR spectra are in accordance with the previous report of the compound.[1]

Hydrochlorination of olefin 15 to compound 4. A solution of olefin **15** (1 g, 1 equiv) and iron (III) chloride (1.35 g, 2 equiv) in water (85 mL) at 4-5 °C was deoxygenated with N_2. Phenylsilane (3.2 mL, 6 equiv) was added followed by MeCN (1.5 mL). The reaction was allowed to stir to room temperature for 15 h. More of iron (III) chloride (0.676 g, 1 equiv) was added, stirred at rt for 24 h. More of phenylsilane (0.1 mL, 0.2 equiv) was added and the mixture was stirred for 5 h at rt. The reaction mixture was then diluted with 1:1 MTBE:hexanes (40 mL), stirred rigorously and allowed to settle. The aqueous layer was separated, treated with EDTA- disodium salt dihydrate (4.65 g, 3 equiv), saturated with NaCl, and the product was extracted with 2-MeTHF (3×50 mL). The combined organic extracts were washed with brine and concentrated *in vacuo*, replacing 2-MeTHF with iPrOAc. The product was crystallized from iPrOAc-MTBE, filtered, and dried to provide 950 mg (82% yield) of compound **4** as a solid. The NMR spectra are in accordance with the previous report of the compound.[2]

Preparation of 16 by methylation of 12 with MeMgBr / $ZnCl_2$. To a dry round bottom flask was charged dry 2-MeTHF (30 mL), 2.5 equiv $ZnCl_2$ (16.0 mL, 30.5 mmol, 1.9 M in 2-MeTHF) and mixture was cooled to 0 - 5 °C under N_2. To the cold mixture was added 5.0 equiv 3.0 M MeMgBr in Et_2O (20.3 ml, 60.9 mmol) slowly *via* syringe while maintaining the internal temperature below 10 °C. After the Grignard addition, the slurry was allowed to warm to rt and aged for 1 h. The slurry was then cooled again to 0-2°C and a slurry of 2'-ketone **12** (5.0 g, 12.18 mmol) in dry toluene (100 mL) was added slowly while maintaining the internal temperature below 10 °C. The reaction mixture was then aged at rt overnight. The reaction after 24 h age at rt was determined by HPLC to be at 98.3% conversion with 21 : 1 dr in favor of the desired diastereomer **16**. The reaction mixture was cooled to 10 °C and quenched with 1 N HCl (75 mL) and aged at rt for 1 h. The layers were separated and the aqueous layer was back extracted with additional toluene (25 mL). The combined organic layer was washed with brine (25 mL). The organic layer was dried over $MgSO_4$, filtered and the cake rinsed with toluene (2×10 mL) to afford 4.89 g (94% combined assay yield) as a 21 : 1 dr of **16 : 17**, The assay yield of desired diastereomer **16** was 4.68 g (90%). The reaction was monitored by HPLC (Ascentis Express C18, 4.6×150 mm, 2.7 µm; B = CH_3CN, A = 0.1% H_3PO_4 in H_2O; 10% B to 90% B over 10 min, hold at 90% B for 5 min, post time = 4 min; flow rate = 1.0 mL/min; injection volume = 5 µL; detection = 261 nm; retention times: 9.98 min & 11.35 min = hydrate & keto of starting material (**12**), 11.19 min = desired diastereomer **16** and 10.37 min = undesired diastereomer **17**. Analytical sample of **16** was obtained *via* flash chromatography purification (EtOAc/hexanes). Compound **16**: ^{1}H NMR ($CDCl_3$, 500 MHz): δ 9.58 (s, 1H), 7.68 (d, J = 8.2 Hz, 1H), 6.04 (s, 1H), 5.64 (dd, J = 8.2, 1.6 Hz, 1H), 4.90 (d, J = 3.2 Hz, 1H), 4.59 (dd, J = 11.9, 7.0 Hz, 1H), 4.33 (dd, J = 11.9, 4.0, Hz, 1H), 4.10 (ddd, J = 7.1, 4.0, 3.2, Hz, 1H), 1.42 (s, 3H), 1.25 (s, 9H), 1.24 (s, 9H). ^{13}C NMR ($CDCl_3$, 125 MHz,): δ 178.44, 177.75, 164.22, 150.93, 142.81, 101.44, 89.50, 81.16, 79.77, 79.22, 63.51, 39.11, 39.04, 27.37, 27.25, 19.63. HRMS $[M+H]^+$ for $C_{20}H_{31}N_2O_8$ calc'd 427.2080; found 427.2092.

Preparation of 16 by methylation of 12 with MeMgBr / $MnCl_2$. To a vessel under nitrogen was charged anhydrous anisole (450 kg) and anhydrous manganese dichloride (particle size < 100 μm) (89 kg, 706 mol, 3.2 equiv, pre-milled to small particle size of D90 = 32 μm). The slurry was cooled to 0 °C and methyl magnesium bromide (275 kg, 3.0 M in 2-MeTHF, 657 mol, 3 equiv) was slowly charged while keeping at 0 °C. The mixture was agitated for 6 h (note: aging is important for generation of MeMnCl) at 20-30 °C and then cooled to -15 °C followed by charge of 2-MeTHF (388 kg). Solid ketone **12** was charged at -15 – 0 °C slowly over 12 h (note: lower temperature and slow charge are important for good *d.r.*), in total of 91.5 kg solid (90.0 kg after correcting for wt%, 219 mol). After aging for additional 3 h, HPLC analysis indicated complete consumption of ketone **12** (0.1% left) and the product *d.r.* of 95-97:5-3 (19-32 : 1). The batch was inverse quenched into a mixture of 35% HCl (123 kg), water (1100 kg) and 2-MeTHF (150 kg) 0-10 °C. The reaction vessel was rinsed with additional 2-MeTHF (360 kg) and combined into the quench. After layer cut, the organic layer was washed with 15% brine (500 kg), then 20% brine (500 kg). The organic layer was then concentrated under vacuum to about 8X volume affording 764 kg of solution at 11.4% wt of the **16**, corresponding to 81.7 kg desired stereoisomer **16** or 93% yield. This solution was used directly in the next step.

Formation of anhydrouridine 19 from alcohol 16. To a crude solution of alcohol **16** (9.37 g assay, 22.0 mmol, 1.0 equiv) in anisole-2-Me-THF (~55 mL total volume, <30 wt% Me-THF) obtained in the previous step, was added 37% aq HCl (0.018 mL, 1 mol%) and the mixture was warmed to 75 °C. *N,O*-Bis(trimethylsilyl)acetamide (13.4 g, 3.0 equiv) was added slowly over 1 h and stirred for additional 8 h at 75 °C. The reaction temperature was adjusted to 60 °C and MeOH (100 mL) was added followed by DBU (2.51 g, 0.75 equiv). After 90 min at 65 °C, the batch was seeded and stirred for additional 10 h. The batch was cooled to <40 °C, concentrated to 75 mL volume, solvent-switched at constant volume with 75 mL iPrOH, and cooled to 10 °C. The slurry was filtered and the cake was washed with 1:1 mixture of anisole/iPrOH (30 mL) and iPrOH (90 mL). The cake was dried under nitrogen to provide anhydrouridine **19** (4.62 g, 87%). The NMR spectra of compound **19** are in accordance with the previous report of the compound.[3] Analytical sample of the TMS ether impurity **20** could be isolated by flash chromatography on silica gel using hexane-ethyl acetate as eluent. ^{1}H NMR ($CDCl_3$, 500 MHz): δ 9.68 (s, 1H), 7.59 (d, *J* = 8.2 Hz, 1H), 6.03 (s, 1H), 5.73 (dd, *J* = 8.2, 1.9 Hz, 1H), 4.90 (d, *J* = 1.6 Hz, 1H), 4.62 (dd, *J* = 11.1, 8.2 Hz, 1H), 4.16 (dd, *J* = 11.1, 5.3 Hz, 1H), 4.07-4.13 (m, 1H), 1.36 (s, 3H), 1.24 (s, 9H), 1.22 (s, 9H), 0.16 (s, 9H). ^{13}C NMR ($CDCl_3$, 125 MHz,): δ 178.10, 177.16, 163.61, 150.82, 142.69, 100.69, 89.32, 81.52, 81.42, 79.01, 63.54, 38.82, 38.78, 27.10, 27.03, 18.43, 1.95. HRMS $[M+H]^+$ for $C_{23}H_{38}N_2O_8Si$ calc'd 499.2476; found 499.2470.

Hydrochlorination of anhydrouridine 19 to compound 4. To a slurry of anhydrouridine **19** (900 g, 1.0 equiv) in 1,2-dimethoxyethane (9 L) and *N,N*-dimethylformamide (137 g) was added dichlorodimethylsilane (1.45 kg, 3.0 equiv) over 1 h at rt. The mixture was heated at 40 °C for 1 h, then at 70 °C for 10 h. The mixture was concentrated *in vacuo* at 40 °C to 7.2 L volume, reconstituted with 1,2-dimethoxyethane (2.7 L), and continuously distilled with a mixture of 1,2-dimethoxyethane (6.3 L) and water (67 mL, 1.0 equiv), maintaining 10 L total volume. The mixture was concentrated *in vacuo* to 4.5 L volume at 45 °C, cooled to 20 °C, and quenched with water (1.7 L) over 1 h. The bottom layer was separated and deoxygenated with N_2. $FeCl_3•(H_2O)_6$ (506 g, 0.5 equiv) was added at 20 °C and the solution was deoxygenated with N_2. 1,1,3,3-Tetramethyldisiloxane (252 g, 0.5 equiv) was deoxygenated with N_2 and added to the reaction mixture over 30 min at 25 °C and the mixture was stirred for 12 h at 25 °C. Added 2-MeTHF

(5.4 L), water (2.7 L), and Na_2SO_4 (1.42 kg), mixed and then cut away the aqueous layer. Added water (1.8 L) and Na_2SO_4 (0.504 kg), mixed, and then cut away the aqueous layer. Added CUNO-5 carbon (45 g), filtered, and rinsed the cake with 2-MeTHF (2.9 L). The filtrate was concentrated to 6.3 L volume at 55 °C with vacuum distillation, then distilled with methyl isobutyl ketone (4.5 L) at 55 °C and 6.3 L constant volume. The batch was seeded and distillation with methyl isobutyl ketone (8.1 L) was continued at 55 °C and 6.3 L constant volume. *n*-Heptane (1.8 L) was added over 2 h at 55 °C, the slurry was cooled to 20 °C and filtered. The cake was washed with a 5:2 mixture of methyl isobutyl ketone and *n*-heptane (3×1.8 L) and dried with nitrogen sweep to provide 881 g (85% yield) of compound **4** as a solid. The NMR spectra are in accordance with the previous report of the compound.[2]

Preparation of alanine ester 21b solution. To a slurry of D-alanine (22 kg, 1.0 equiv) in iPrOH (123 L) was added TMS-Cl (40.2 kg, 1.5 equiv) over 30 min keping the temperature below 50 °C. The mixture was stirred at 70 °C for 12 h. Cooled to 45 °C and Et_3N (2.6 kg, 0.11 equiv) was added to pH 3-5. The batch was concentrated to 88 L volume while maintaining batch temperature at 50 °C (~100 torr). Added iPrOAc (44 L) and solvent switched at constant 130 L volume at 50 °C using more iPrOAc (450 L). Added iPrOAc (79 L) followed by Et_3N (25.1 kg, 1.0 equiv) to pH>9. The slurry was cooled to 0 °C, filtered, and the cake was washed with iPrOAc (123 L). The combined filtrates were concentrated to 50-60 wt% using wiped film evaporator at 40 mm Hg and 40 °C on the jacket. The concentrated product was then distilled using wiped film evaporator at 10 mmHg and 65 °C on the jacket to provide 29.2 kg (80%) assay yield of compound **21b** as ~50 wt% solution in iPrOAc that was stored at +5 °C and used directly in the next step.

Preparation of chlorophosphoramidate 22 solution. In the first vessel, phenyl dichlorophosphate (22.4 kg, 1.0 equiv) was dissolved in dry iPrOAc (76 L) and the solution was cooled to -20 °C. In the second vessel, a mixture of alanine ester **21b** (1.02-1.05 equiv), prepared above, and Et_3N (11.3 kg, 1.05 equiv) was cooled to -20 °C and then added over 2 h to the first vessel at < -10 °C. After 1 h at < -10 °C, the slurry was filtered under nitrogen and the cake was washed with dry iPrOAc (76 kg). The filtrates were combined and concentrated to ~35 wt% using vacuum distillation at 20 °C to provide 29.2 kg (90%) assay yield of **22** that was used directly in the next step.

Preparation of uprifosbuvir 1. A mixture of compound **4** (20 g, 1.0 equiv), dimeric chiral imidazole carbamate catalyst **24**[4] (0.96 g, 3 mol%), 2,6-lutidine (12.6 mL, 1.5 equiv), and 1,3-dioxolane (120 mL) was cooled to -10 °C. The chlorophosphoramidate **22** solution in iPrOAc prepared above (1.2 equiv) was added over 1 h maintaining the temperature at -10 °C. The mixture was stirred at -10 °C for 24 h. iPrOAc (80 mL) was added and the mixture was warmed to 0 °C. A 10% aq solution of $NaHSO_4$ (40 mL) was added and the mixture was stirred at 30 °C for 1 h. The lower phase was cut away. An aqueous solution of 5% $NaHCO_3$ and 5% Na_2SO_4 (60 mL) was added and the mixture was stirred at 50 °C for 10 h. The lower phase was cut away. The organic phase was washed with 10% aq NaCl (60 mL) at 50 °C and then concentrated under vacuum at 50 °C to 160 mL volume. While maintaining the batch volume at 160 mL, the mixture was distilled with iPrOH (400 mL). The mixture was warmed to 70 °C, filtered hot, and the filtrate was cooled to 55 °C, seeded, and stirred at 55 °C for 2 h. Added *n*-heptane (160 mL), cooled the batch to 20 °C, filtered, washed the cake with 1:1 iPrOH-*n*-heptane (3×80 mL), *n*-heptane (120 mL), and dried under nitrogen

sweep to provide 34.7 g (88% yield) of uprifosbuvir **1**. The NMR spectra are in accordance with the previous report of the compound.[5]

Isolation and characterization of 11·BF_3 complex

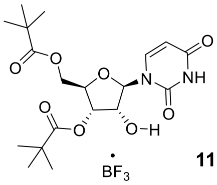

11 · BF_3 complex

After completing preparation of **11·BF_3** complex as a slurry in toluene as described above, the mixture was filtered, washed with toluene and vacuum dried at rt under N_2 affording **11·BF_3** complex as a white solid. HRMS (m/z): calculated for $[C_{19}H_{27}BF_3O_8]^-$, 479.1818; observed 479.1839.

X-Ray powder diffraction data of 11·BF_3 complex

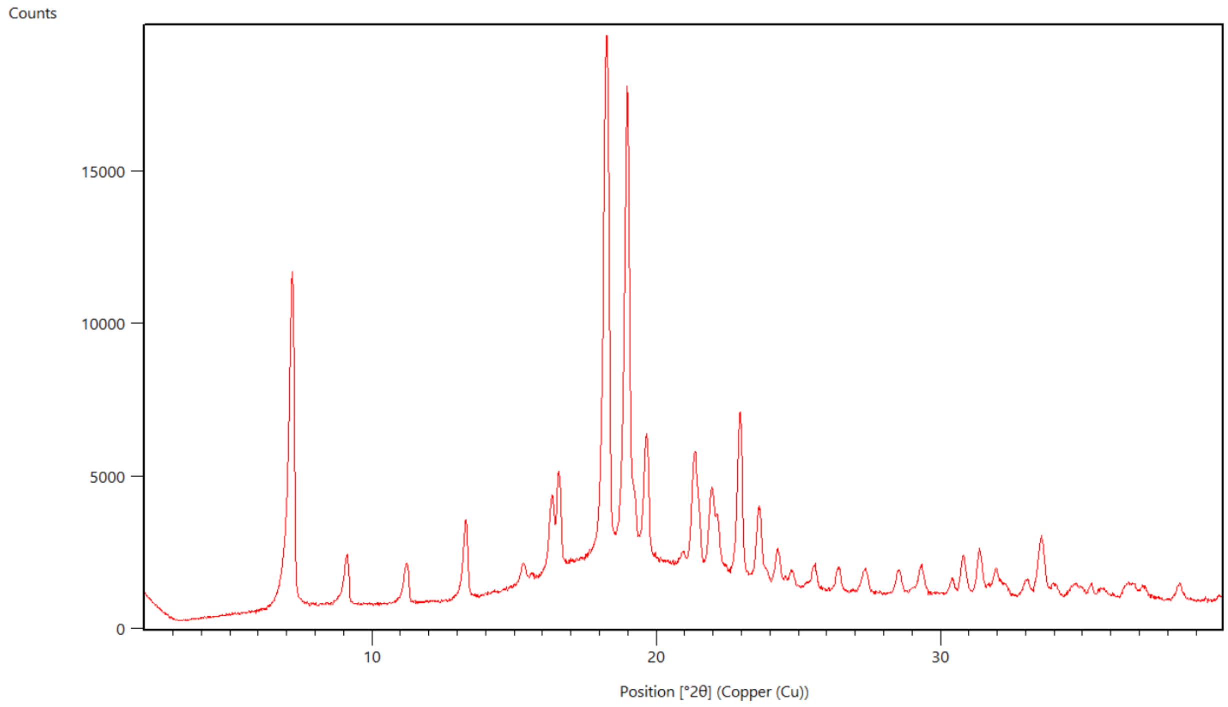

HRMS [M-H]$^-$ of 11·BF$_3$

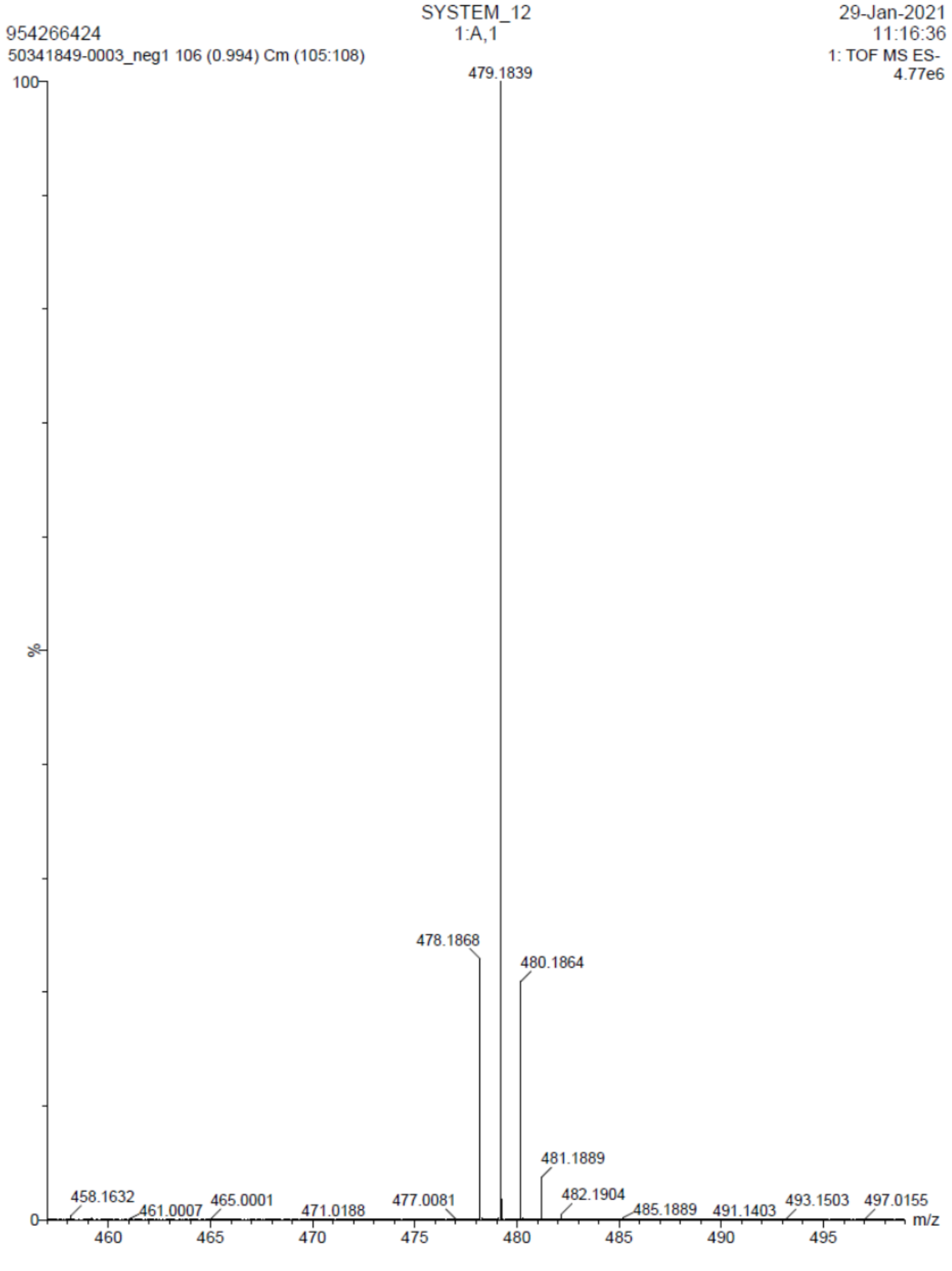

^{18}O-Labeling experiments for the cyclization of alcohol 16 into anhydrouridine 18

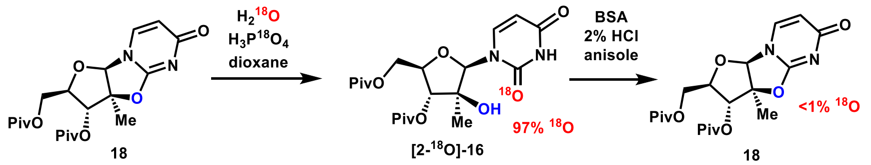

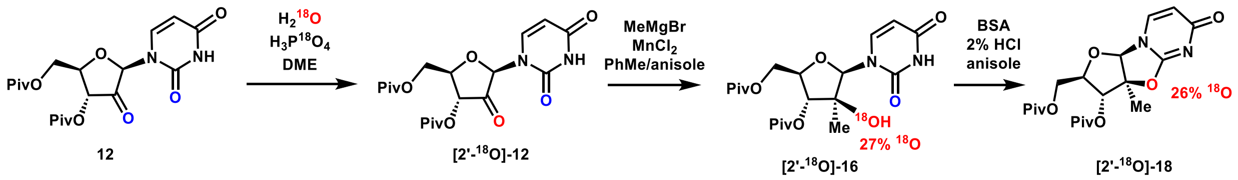

Preparation of labeled alcohol [2-^{18}O]-16. Anhydrouridine **18** (449 mg, 1.1 mmol) and 1,4-dioxane (1.078 ml) were added to a 20 mL vial followed by [^{18}O]-water (77 mg, 3.85 mmol) and [^{18}O]-phosphoric acid (128 mg, 1.21 mmol). Heated the mixture for 4 h to 65 °C. Added 1 mL MeOH and then 4 mL water over 30 min and stirred for 1 h while cooling to 5 °C. Filtered off the crystals, washed the cake with 5 mL of 2:1 MeOH-water, and dried under N_2 stream to give the crude desired compound [2-^{18}O]-**16**. This material was recrystallized by dissloving it in 3 mL MeOH followed by addition of 3 mL water over 20 min. The slurry was filtered and washed with 5 mL of 2:1 MeOH-water, and dried under N_2 stream to give product. This material was further recrystallized by dissolving in 3 mL of CPME at 80 °C followed by addition of 3 mL heptane over 20 min and cooling to 20 °C for 1 h. The slurry was filtered and washed with 5 mL 2:1 hexane-MTBE, and dried under N_2 stream to give the purified [2-^{18}O]-**16** (300 mg, 0.700 mmol, 64% yield). LCMS showed 97% incorporation of ^{18}O.

Preparation of labeled alcohol [2'-^{18}O]-16. In a 20 mL vial, ketone **12** (0.821 g, 2 mmol), 5 mL DME, [^{18}O]-water (0.100 g, 5.00 mmol) and [^{18}O]-phosphoric acid (1.06 mg, 10.00 µmol) were heated to 65 °C for 10 min. Mixture was evaporated to dryness under N_2 stream, then flushed twice with 5 mL DME. Then 5 mL DME and [^{18}O]-water (0.100 g, 5.00 mmol) were added and the mixture was heated to 65 °C for 10 min. The mixture was evaporated to dryness under N_2 stream, then flushed twice with 5 mL DME. Then again 5 mL DME and [^{18}O]-water (0.100 g, 5.00 mmol) were added and the mixture was heated to 65 °C for 10 min. The mixture was evaporated to dryness under N_2 stream, then flushed twice with 5 mL DME. Then again 5 mL DME and [^{18}O]-water (0.100 g, 5.00 mmol) were added and the mixture was heated to 65 °C for 10 min. The mixture was evaporated to dryness under N_2 stream, then flushed twice with 5 mL DME. The residue was 783 mg. This crude ketone [2-^{18}O]-**12** was taken directly into the next step.

Manganese(II) chloride (0.717 g, 5.70 mmol) was stirred magneticaly under N_2 overnight. 4 mL anisole was added followed by methylmagnesium bromide (3.0 M, 2.00 ml, 6.00 mmol) and the mixture was stirred for 3 h at 22 °C to

prepare the MeMnX reagent. The crude ketone [2-^{18}O]-**12** was suspended in 3 mL MeTHF and 0.8 mL anisole. This ketone slurry was added to the MeMnX reagent at -20 °C over 5 min. Mixture was stirred for 1 h then warmed to 20 °C for 30 min. Quenched with 30 mL 1M aq NH_4Cl. Separated the organic phase and washed it with 30 mL brine. Organic phase was dried over $MgSO_4$, filtered and evaporated to dryness. The residue was recrystallized by dissolving it in 4 mL CPME at 80 °C followed by addition of 4 mL heptane over 20 min and cooling to 20 °C for 1 h. The slurry was filtered and washed with 5 mL 2:1 hexane-MTBE, and dried under N_2 stream to give the desired alcohol [2'-^{18}O]-**16**. LCMS showed 27% incorporation of ^{18}O.

The cyclization reactions with the two differently labeled alcohols [2-^{18}O]-**16** and [2'-^{18}O]-**16** were performed using the procedure described for the unlabeled alcohol **16** except that 2 mol% of HCl was used as the catalyst. The incorporation of the ^{18}O label in the product anhydouridine **18** was determined by LCMS.

Band-selective CLIP-HSQMBC experiment to detect the isotope shift for the carbon bearing the ^{18}O-label in compound 16

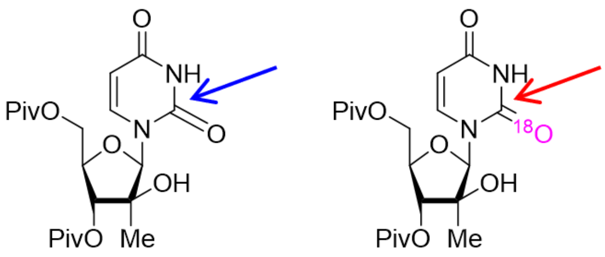

25 : 75

1:3 mixture of unlabeled **16** and labeled compound [2-^{18}O]-**16** (97% ^{18}O).

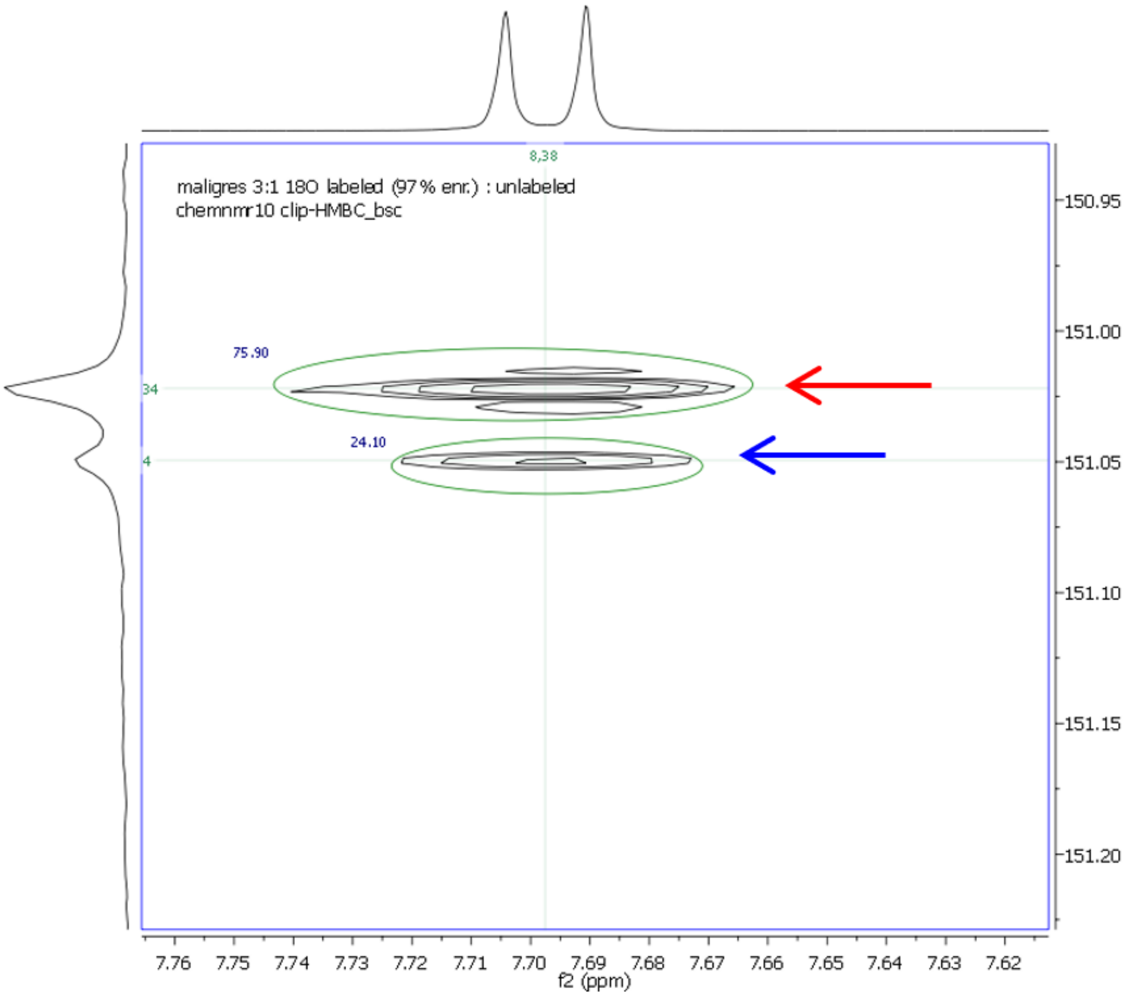

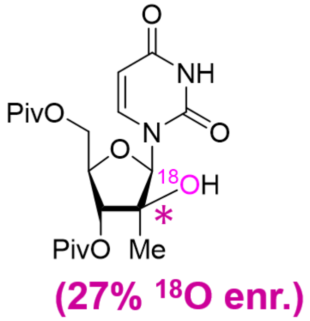

Labeled compound [2’-^{18}O]-**16** (27% ^{18}O).

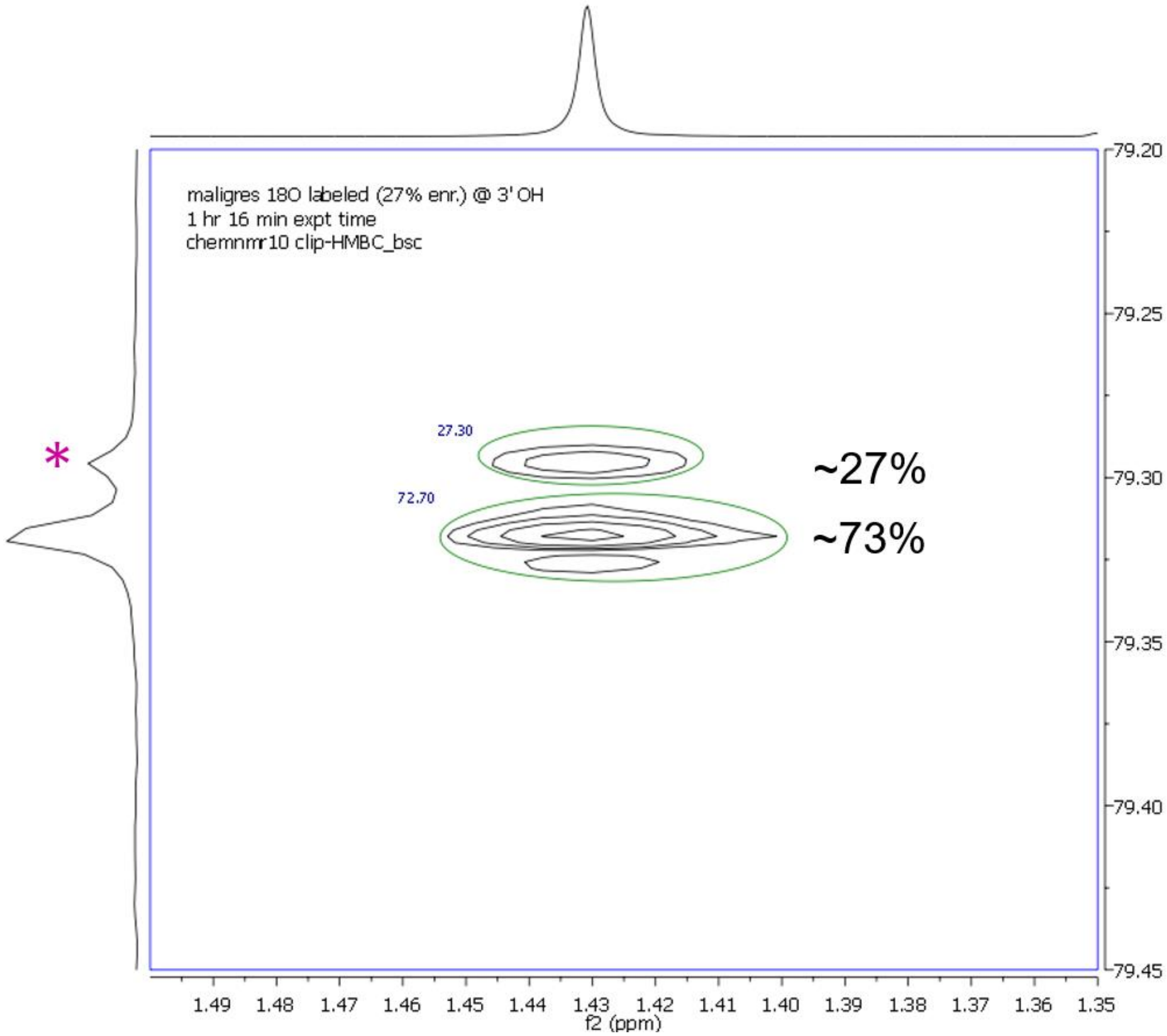

NMR Spectroscopy Data

^{1}H and ^{13}C NMR Spectra of 10/11:

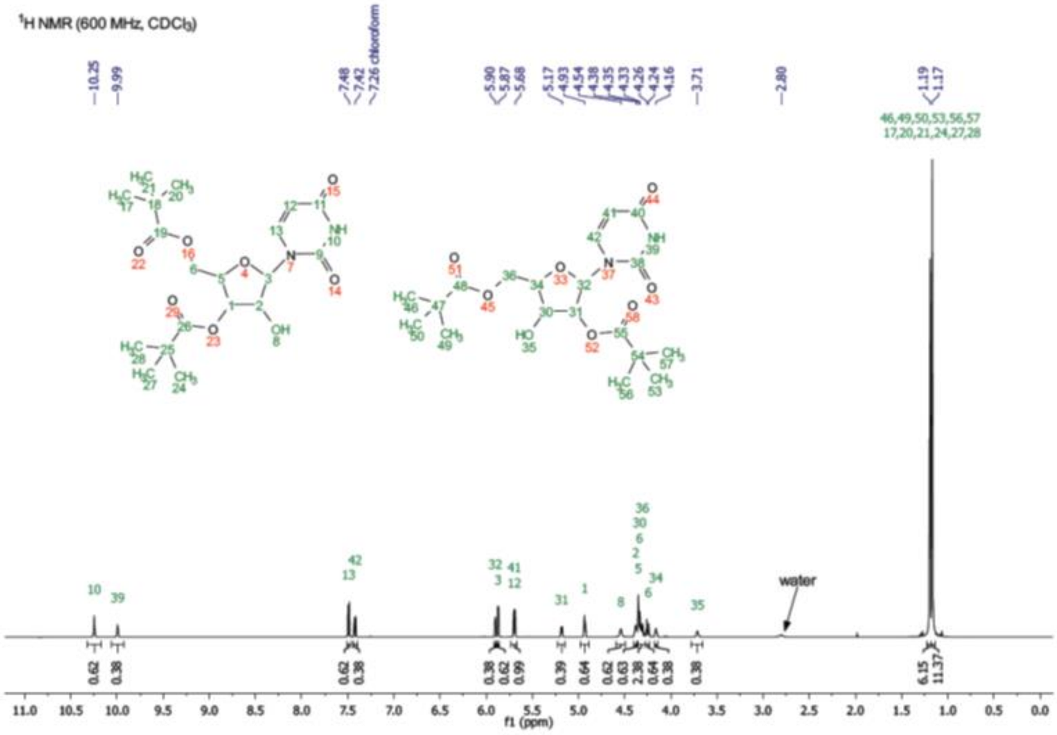

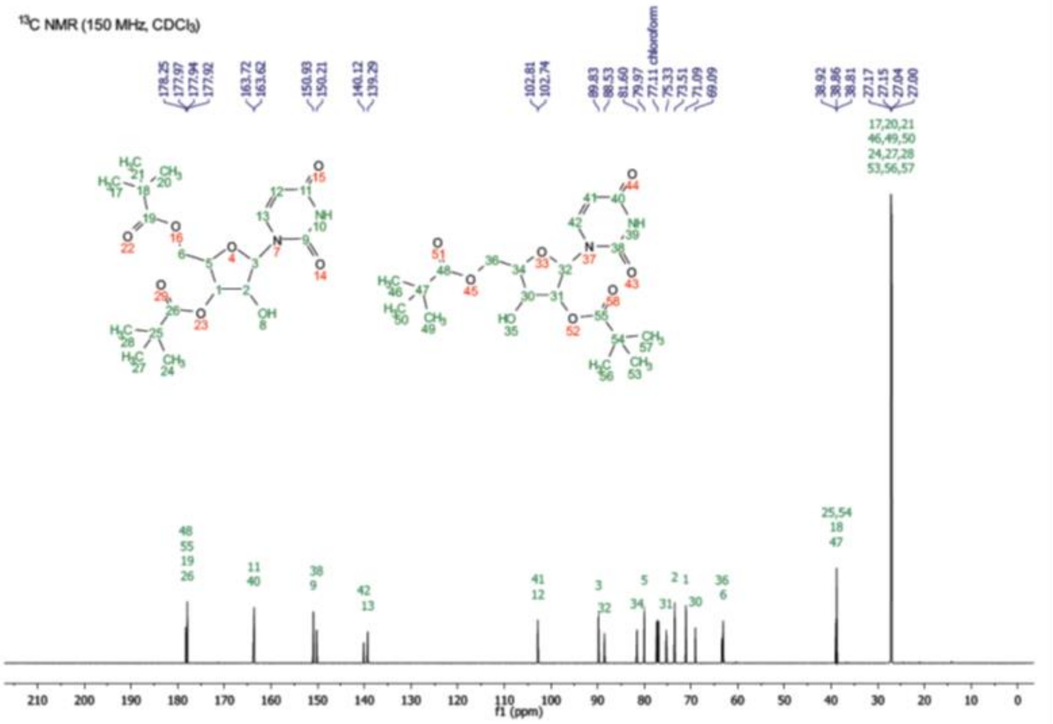

^{1}H NMR Spectrum of 10:

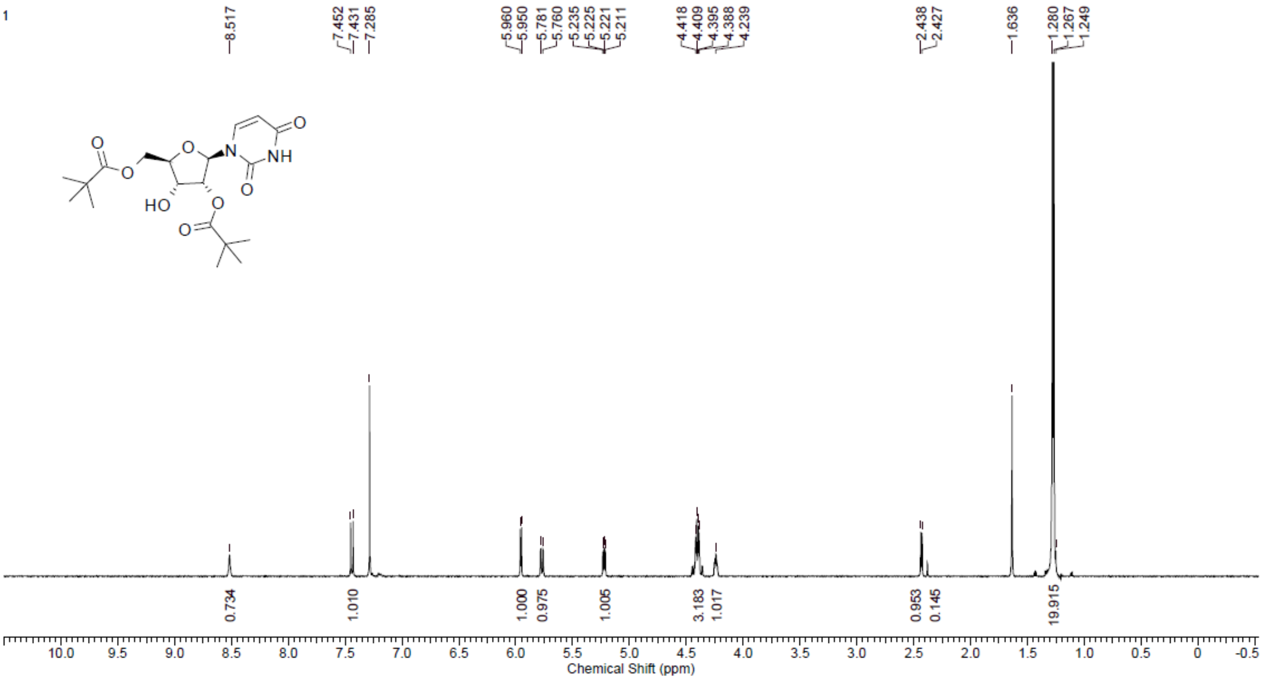

^{1}H and ^{13}C NMR Spectra of 12:

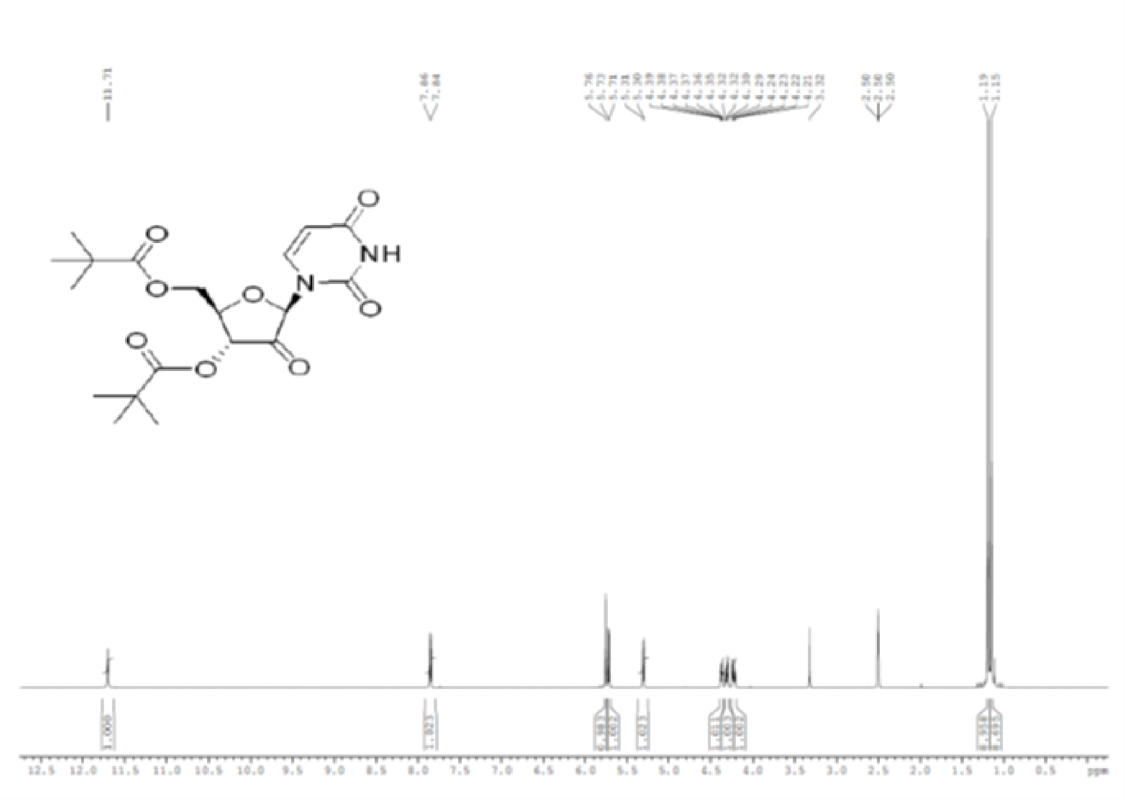

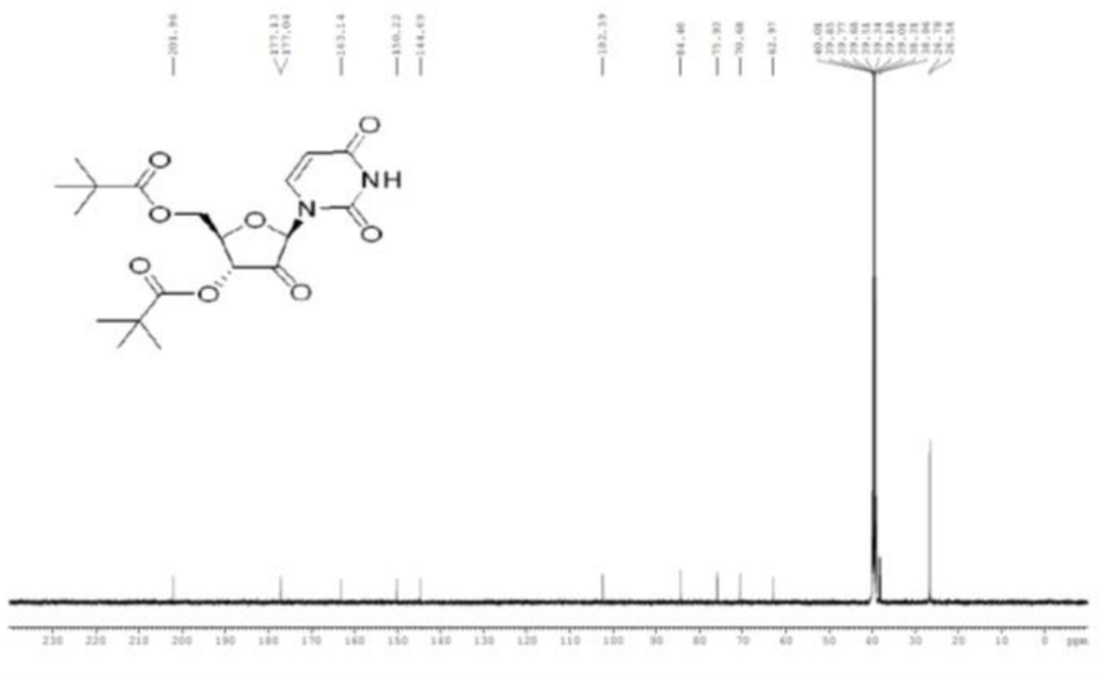

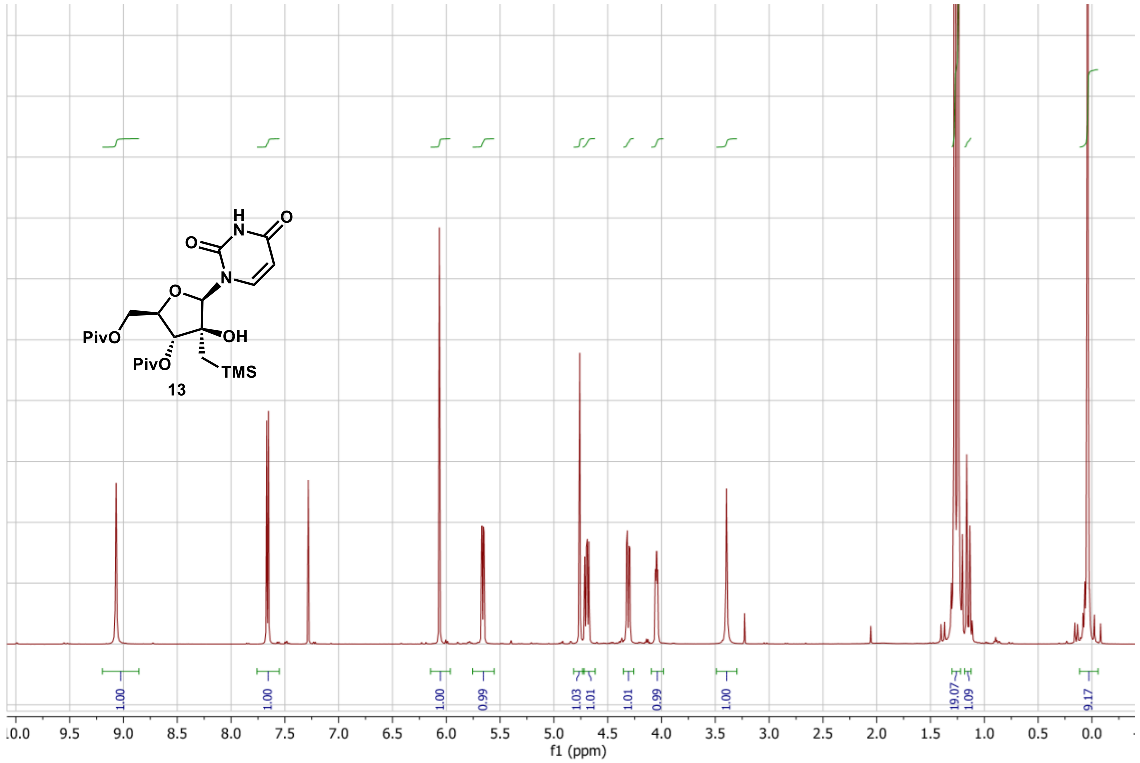
H
N
O
O
O
N
PivO
OH
PivO
TMS
13
1.00
1.00
1.00
0.99
1.03
1.01
1.01
0.99
1.00
19.07
1.09
9.17
f1 (ppm)

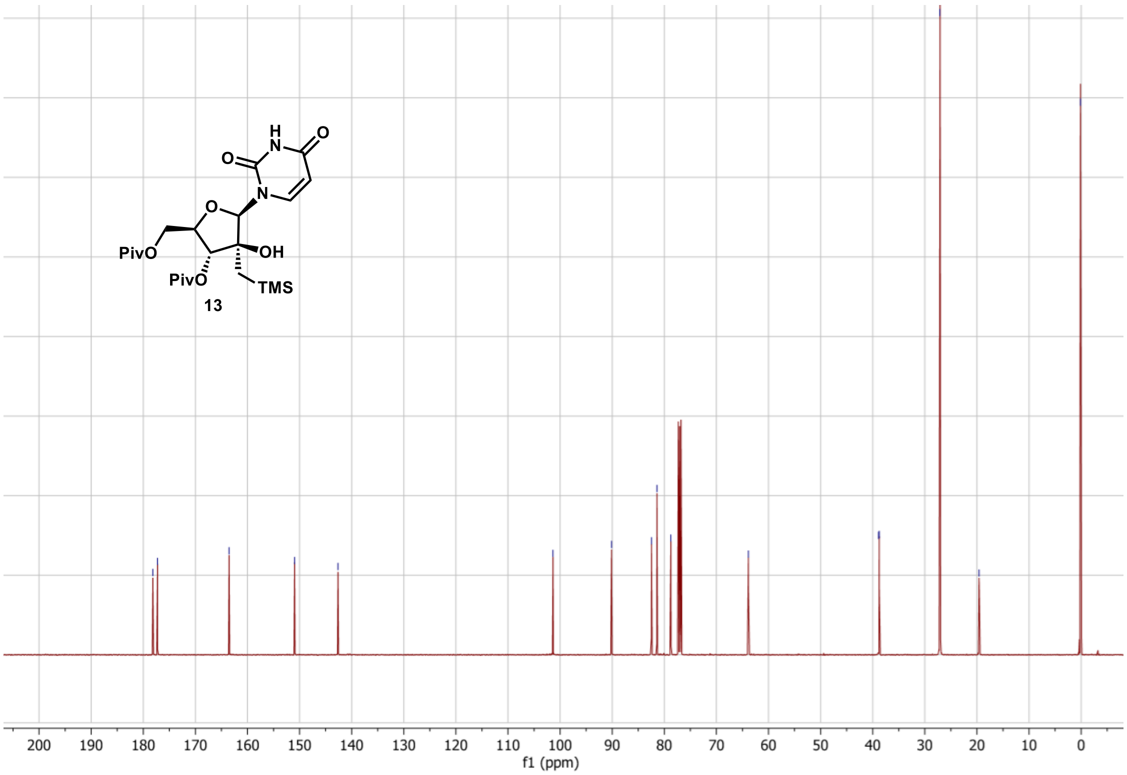
H
N
O
O
O
N
PivO
OH
PivO
TMS
13
f1 (ppm)

^{1}H and ^{13}C NMR Spectra of 16:

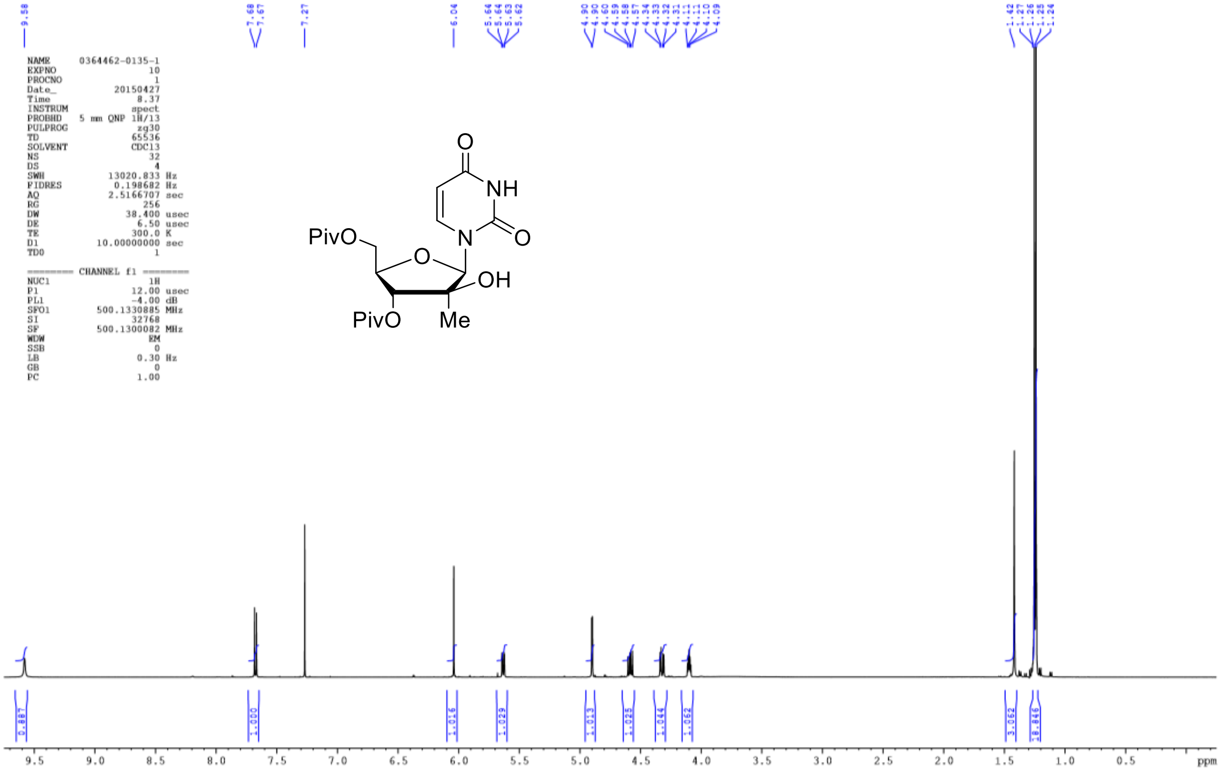

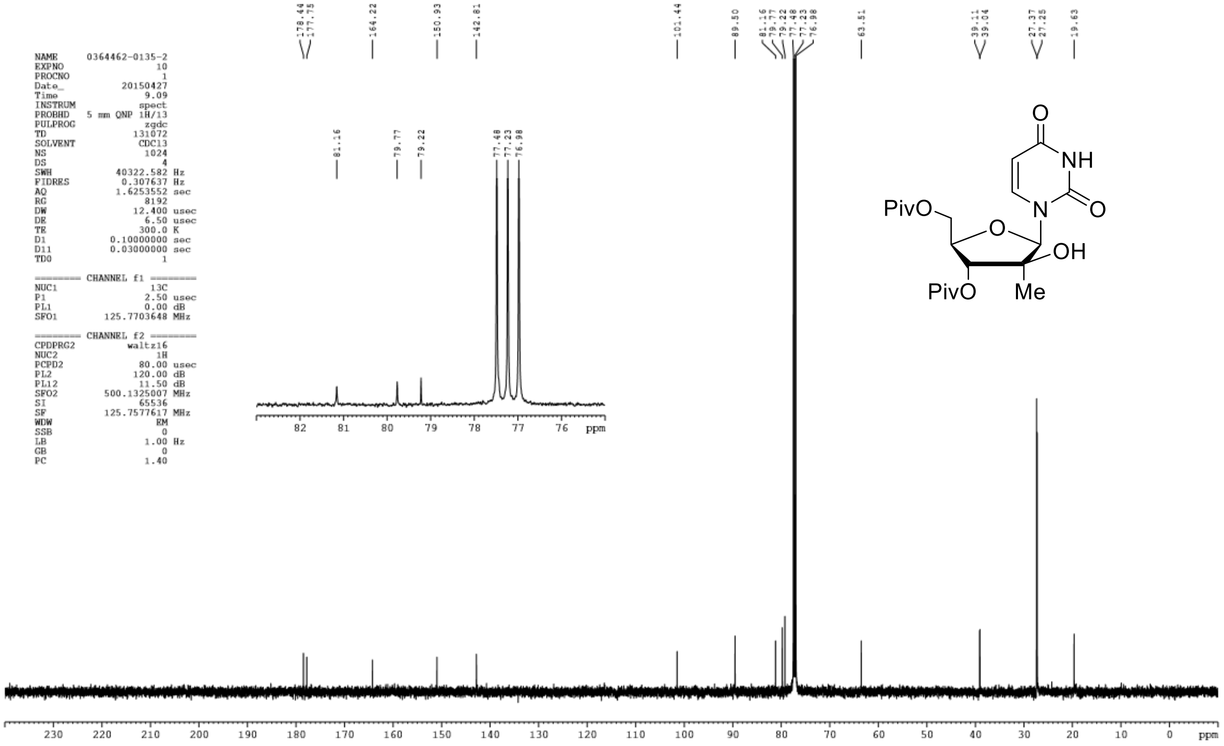

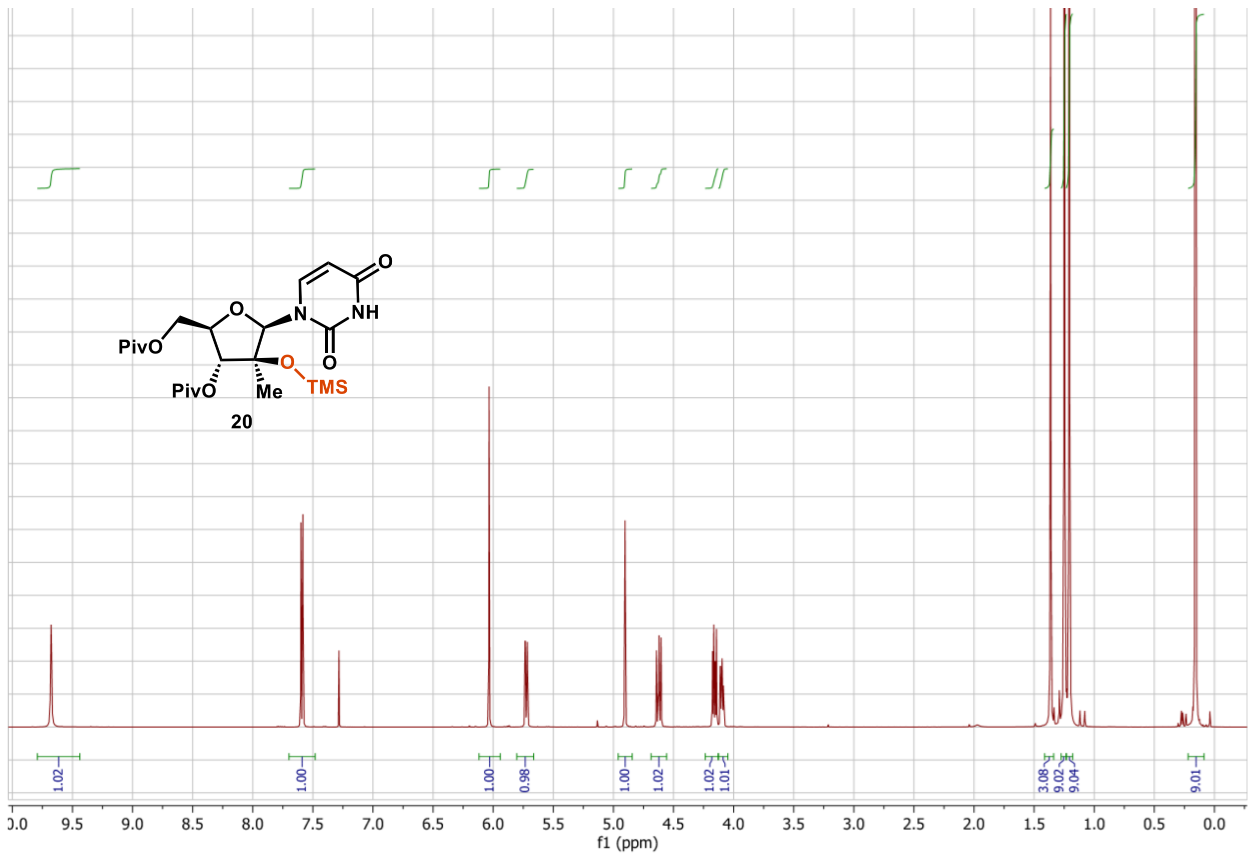
PivO
PivO
O
N
NH
O
O
Me
O
TMS
20
1.02
1.00
1.00
0.98
1.00
1.02
1.02
1.01
3.08
9.02
9.04
9.01
f1 (ppm)

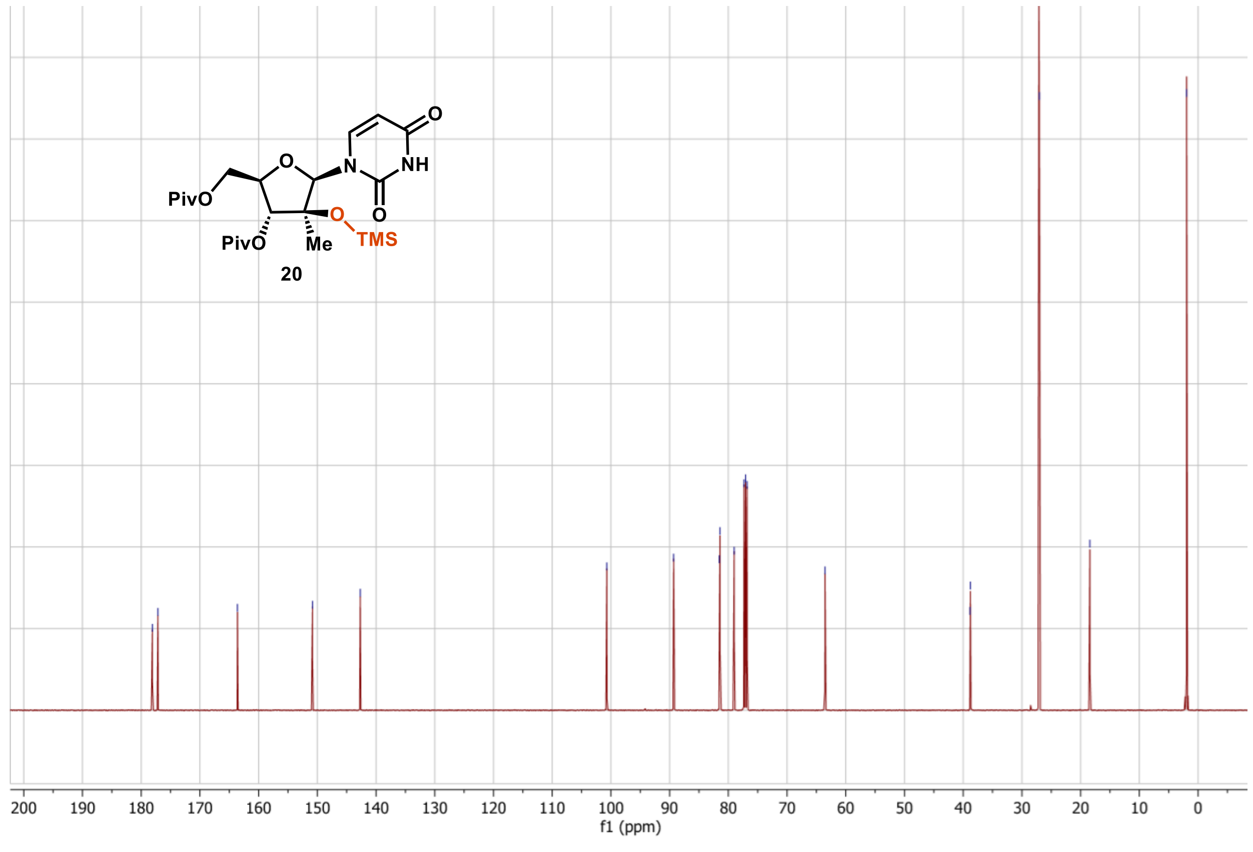
PivO
PivO
O
N
NH
O
O
Me
O
TMS
20
f1 (ppm)

[1] S. Lemaire, I. Houpis, R. Wechselberger, J. Langens, W. A. A. Vermeulen, N. Smets, U. Nettekoven, Y. Wang, T. Xiao, H. Qu, R. Liu, T. H. M. Jonckers, P. Raboisson, K. Vandyck, K. M. Nilsson and V. Farina, *J. Org. Chem.* 2011, **76**, 297–300.

[2] G. Wang, N. Dyatkina, M. Prhavc, C. Williams, V. Serebryany, Y. Hu, Y. Huang, J. Wan, X. Wu, J. Deval, A. Fung, Z. Jin, H. Tan, K. Shaw, H. Kang, Q. Zhang, Y. Tam, A. Stoycheva, A. Jekle, D. B. Smith and L. Beigelman, *J. Med. Chem.* 2019, **62**, 4555−4570.

[3] A. M. Hyde, R. Calabria, R. Arvary, X. Wang and A. Klapars, *Org. Process Res. Dev.,* 2019, **23**, 1860−1871.

[4] D. A. DiRocco, Y. Ji, E. C. Sherer, A. Klapars, M. Reibarkh, J. Dropinski, R. Mathew, P. Maligres, A. M. Hyde, J. Limanto, A. Brunskill, R. T. Ruck, L.-C. Campeau and I. W. Davies, *Science,* 2017, **356**, 426–430.

[5] Z. Liu, A. Klapars, B. Simmons, A. Bellomo, A. Kalinin, M. Weisel, J. Hill and S. M. Silverman, *Org. Process Res. Dev.,* 2021, **25**, 661-667.
